# Selective Inhibition of 2-Oxoglutarate and 2-Oxoadipate Dehydrogenases by the Phosphonate Analogs of Their 2-Oxo Acid Substrates

**DOI:** 10.3389/fchem.2020.596187

**Published:** 2021-01-12

**Authors:** Artem V. Artiukhov, Alexey V. Kazantsev, Nikolay V. Lukashev, Marco Bellinzoni, Victoria I. Bunik

**Affiliations:** ^1^Faculty of Bioengineering and Bioinformatics, Lomonosov Moscow State University, Moscow, Russia; ^2^Department of Biokinetics, A. N. Belozersky Institute of Physicochemical Biology, Lomonosov Moscow State University, Moscow, Russia; ^3^Faculty of Chemistry, Lomonosov Moscow State University, Moscow, Russia; ^4^Unité de Microbiologie Structurale, Institut Pasteur, CNRS, Université de Paris, Paris, France; ^5^Department of Biochemistry, Sechenov University, Moscow, Russia

**Keywords:** succinyl phosphonate, glutaryl phosphonate, adipoyl phosphonate, OGDH, OADH, DHTKD1, acyl phosphonate complex with 2-oxo acid dehydrogenase, phosphonate analog of 2-oxo acid

## Abstract

Phosphonate analogs of pyruvate and 2-oxoglutarate are established specific inhibitors of cognate 2-oxo acid dehydrogenases. The present work develops application of this class of compounds to specific *in vivo* inhibition of 2-oxoglutarate dehydrogenase (OGDH) and its isoenzyme, 2-oxoadipate dehydrogenase (OADH). The isoenzymes-enriched preparations from the rat tissues with different expression of OADH and OGDH are used to characterize their interaction with 2-oxoglutarate (OG), 2-oxoadipate (OA) and the phosphonate analogs. Despite a 100-fold difference in the isoenzymes ratio in the heart and liver, similar Michaelis saturations by OG are inherent in the enzyme preparations from these tissues ($K_{m}^{OG}$ = 0.45 ± 0.06 and 0.27 ± 0.026 mM, respectively), indicating no significant contribution of OADH to the OGDH reaction, or similar affinities of the isoenzymes to OG. However, the preparations differ in the catalysis of OADH reaction. The heart preparation, where OADH/OGDH ratio is ≈ 0.01, possesses low-affinity sites to OA ($K_{m}^{OA}$ = 0.55 ± 0.07 mM). The liver preparation, where OADH/OGDH ratio is ≈ 1.6, demonstrates a biphasic saturation with OA: the low-affinity sites ($K_{m,2}^{OA}$ = 0.45 ± 0.12 mM) are similar to those of the heart preparation; the high-affinity sites ($K_{m,1}^{OA}$ = 0.008 ± 0.001 mM), revealed in the liver preparation only, are attributed to OADH. Phosphonate analogs of C5-C7 dicarboxylic 2-oxo acids inhibit OGDH and OADH competitively to 2-oxo substrates in all sites. The high-affinity sites for OA are affected the least by the C5 analog (succinyl phosphonate) and the most by the C7 one (adipoyl phosphonate). The opposite reactivity is inherent in both the low-affinity OA-binding sites and OG-binding sites. The C6 analog (glutaryl phosphonate) does not exhibit a significant preference to either OADH or OGDH. Structural analysis of the phosphonates binding to OADH and OGDH reveals the substitution of a tyrosine residue in OGDH for a serine residue in OADH among structural determinants of the preferential binding of the bulkier ligands to OADH. The consistent kinetic and structural results expose adipoyl phosphonate as a valuable pharmacological tool for specific *in vivo* inhibition of the *DHTKD1*-encoded OADH, a new member of mammalian family of 2-oxo acid dehydrogenases, up-regulated in some cancers and associated with diabetes and obesity.

**Graphical Abstract:**
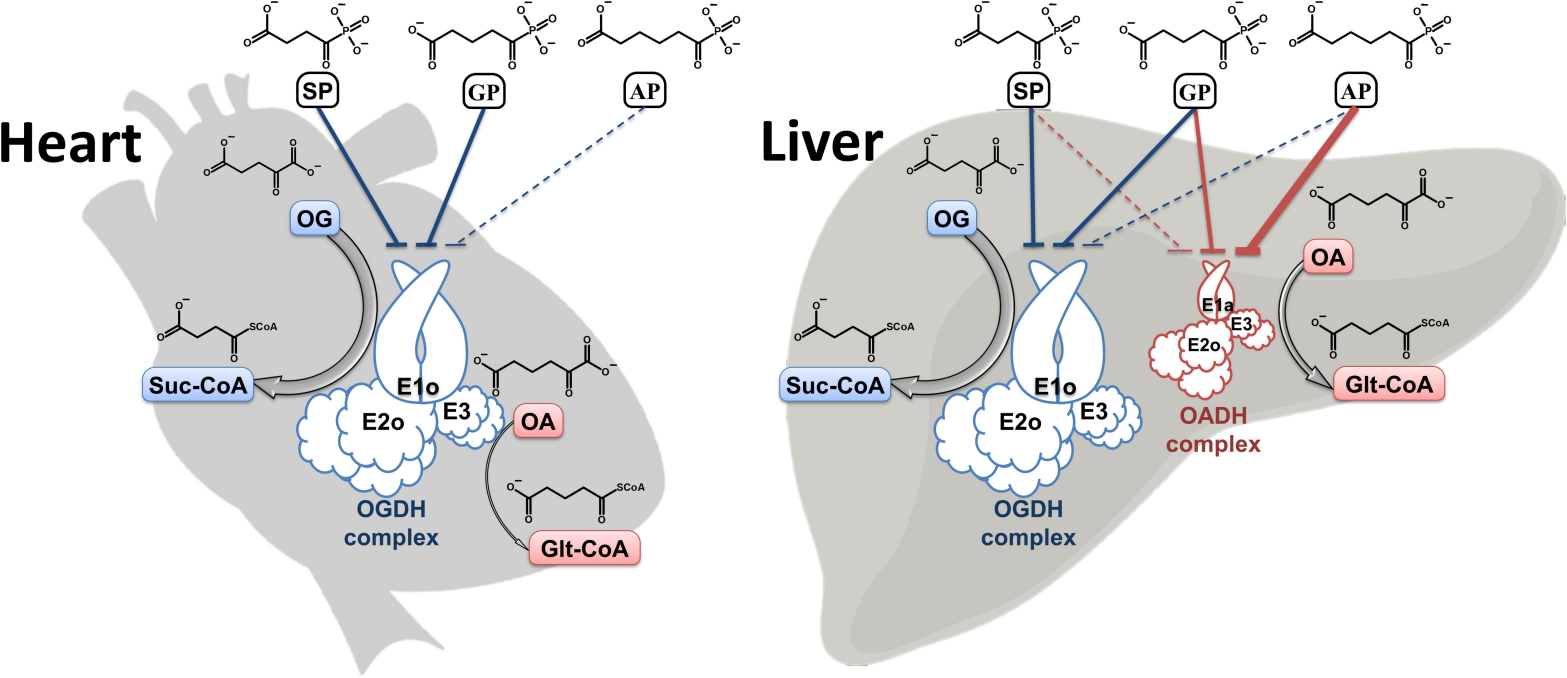


## Introduction

Synthetic phosphonate analogs of pyruvate and 2-oxoglutarate (OG) are specific inhibitors of the reactions, catalyzed by key regulatory enzymes, the thiamine diphosphate (ThDP)-dependent pyruvate dehydrogenase (PDH) and 2-oxoglutarate dehydrogenase (OGDH), respectively (Bunik et al., 2015, 2016; Artiukhov et al., 2016; Bunik, 2017). In these analogs, the phosphonate group replaces the carboxyl group which undergoes the decarboxylation. The interaction of 2-oxo phosphonates with cognate dehydrogenases mostly results in the non-cleavable intermediates, mimicking the enzyme transition state (Kluger and Pike, 1979; Wagner et al., 2019). The tight, yet reversible, binding of the phosphonates to their cognate dehydrogenases allows for specific inhibition of 2-oxo acid dehydrogenase *in vivo* (Bunik et al., 2013; Artiukhov et al., 2016).

**Figure 1:**
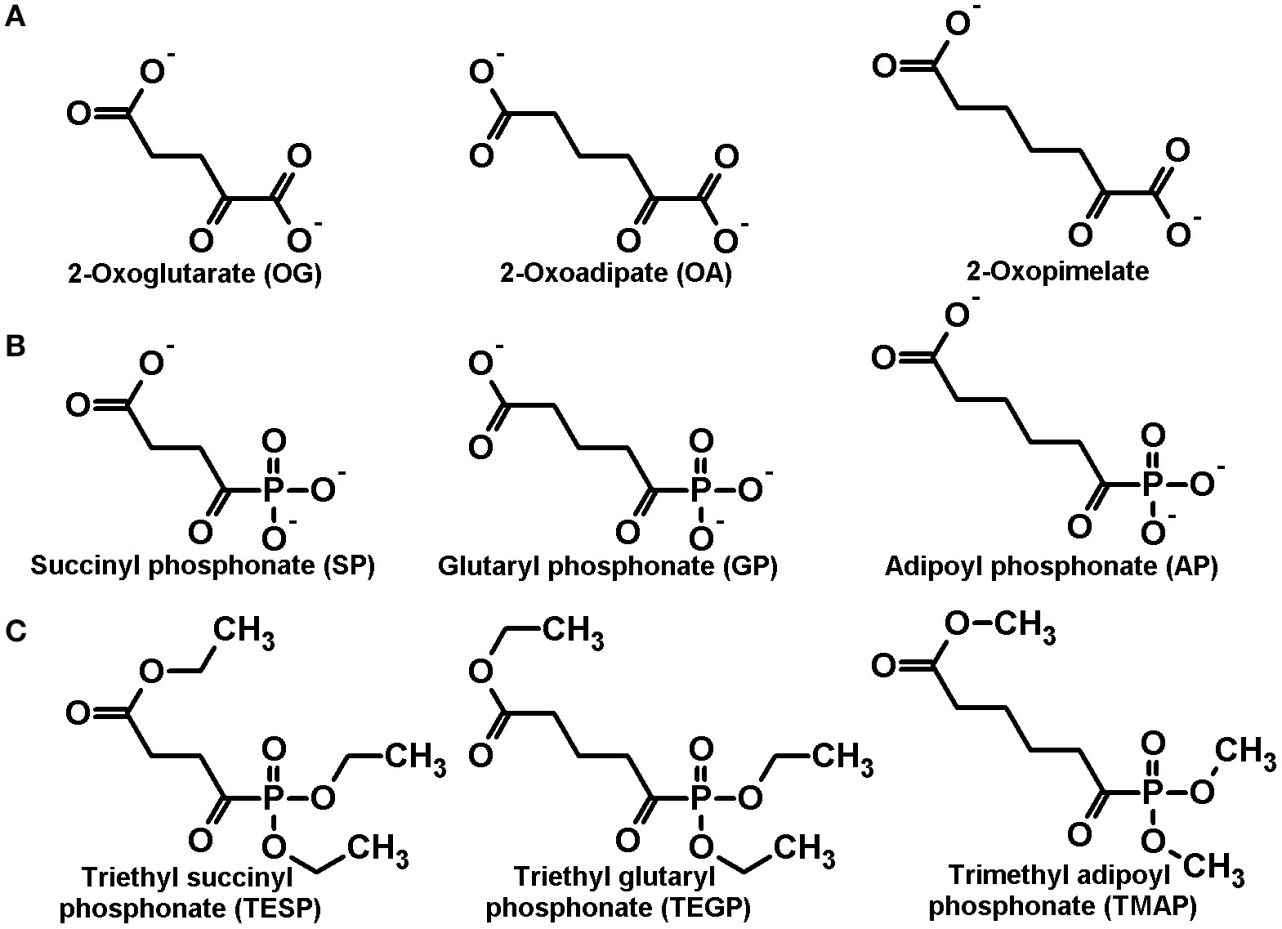
Structures of the dicarboxylic 2-oxo acids **(A)** as well as the corresponding phosphonate analogs **(B)** and their membrane-permeable precursors **(C)**, studied in this work.

The well-known *OGDH-*encoded OGDH is a key enzyme in the mitochondrial tricarboxylic acid (TCA) cycle, whose mutations which impair the function are incompatible with life (Bunik, 2017). In contrast, the physiological significance of the *DHTKD1*-encoded isoenzyme 2-oxoadipate dehydrogenase (OADH), catalyzing oxidative decarboxylation of 2-oxoadipate (OA, Figure 1A), the common intermediate of the lysine and tryptophan catabolism, is much less obvious, as the *DHTKD1* mutations often remain unnoticed. Besides, although OADH is assumed to function as a component of the 2-oxoadipate dehydrogenase multienzyme complex, analogous to the one formed by OGDH, our recent identification of the *DHTKD1*-encoded isoforms of OADH in mammalian tissues has supported our previous prediction from the sequence analysis (Bunik and Degtyarev, 2008), that OADH may also be active in the isolated state, catalyzing the non-oxidative decarboxylation (Boyko et al., 2020). The role of such function in detoxication of aldehydes (Bunik and Fernie, 2009) may underlie association of the dysregulated *DHTKD1* expression with diabetes, obesity and cancer (Lim et al., 2014; Wu et al., 2014; Kiełbus et al., 2015; Plubell et al., 2018; Timmons et al., 2018).

In order to discriminate physiological significance of the reactions catalyzed by the isoenzymes of 2-oxoglutarate dehydrogenase, encoded by the *OGDH* and *DHTKD1* genes, *in vivo*, a homologous series of the phosphonate analogs of dicarboxylic 2-oxo acids (Figure 1B) has been synthesized (Artiukhov et al., 2020), namely the succinyl (SP), glutaryl (GP), and adipoyl (AP) phosphonates. Cellular experiments have shown specific action of the compounds on metabolomes, coinciding with their inhibition of the OGDH and OADH reactions, catalyzed by the partially purified OGDH and OADH. A number of other enzymatic reactions, employing 2-oxo acids, or their structural analogs, are not affected by the phosphonates (Artiukhov et al., 2020).

These promising findings justify a detailed study of the molecular mechanisms underlying the specific action of the phosphonate inhibitors on OADH and OGDH *in vivo*. To develop the most selective inhibitors *in vivo*, our current work aims at comparing the binding of the homologous phosphonate analogs of dicarboxylic 2-oxo acids to OADH and OGDH. We use inhibition kinetics to quantify the binding, and analysis of available structures to identify the molecular origin of the interaction specificity. The quantifications obtained by the kinetic study show a good agreement with the structural data available for the two isoenzymes and the known OGDH complexes with the phosphonates (Wagner et al., 2019). As a result, molecular basis of the selective regulation of the OGDH or OADH isoenzymes by the synthetic phosphonate inhibitors is revealed, providing new knowledge on the directed regulation of the target enzymes in cells and organisms. While SP, whose inhibition of OGDH was first published about three decades ago (Bunik et al., 1992), is by now well-recognized as a specific and efficient inhibitor of OGDH *in vivo*, our development of a similar inhibitor of OADH opens new ways to study the poorly understood biological role of this isoenzyme. Moreover, pharmacological tools to specifically affect the OADH function may be of therapeutic significance, as regulation of the *DHTKD1* expression is observed in a number of pathological conditions, including diabetes, obesity and malignant transformation.

## Materials and Methods

### Reagents

All reagents were of the highest purity available.

Trisodium salts of the succinyl, glutaryl, and adipoyl phosphonates and their ester precursors were synthesized as described elsewhere (Bunik et al., 2005; Artiukhov et al., 2020). Briefly, the trialkyl ester precursors of the phosphonates were prepared by standard Arbuzov reaction of trialkyl phosphite with the corresponding monoalkyl ester chlorides of succinic, glutaric and adipic acids. Trisodium salts of the phosphonates were obtained after the treatment of the corresponding trialkyl esters by bromotrimiethylsilane, followed by alkaline hydrolysis. Subsequent recrystallization from aqueous ethanol provided 95–97% purity salts, according to their ^1^H and ^31^P NMR spectra (Supplementary Figures 1A–D,I,J).

### Enrichment of OGDH and OADH From Rat Tissues and Activity Assays

Enzymes catalyzing OGDH and OADH reactions were enriched from rat hearts and livers using polyethylene glycol precipitation, as previously described (Artiukhov et al., 2020).

The enzyme activities were assayed in the medium (pH 7.0) containing 50 mM MOPS, 1 mM ThDP, 1 mM MgCl_2_, 1 mM CaCl_2_, 1 mM dithiothreitol 0.15 mM Coenzyme A, 0.25 mM NAD^+^ and various concentrations of 2-oxo substrates (0–10 mM OG or OA) and their phosphonate analogs (0–5,000 μM SP, GP, or AP). Reactions were started by addition of the enzyme preparations to the assay media. The amount of enzyme preparations (0.074 mg protein for the preparation from liver and 0.023 mg protein for the preparation from heart) were chosen to induce the lowest reliably measured reaction rate (ΔA_340_ = 0.001/min) at 0.01 mM OA without the phosphonates.

Reaction rates were calculated from linear parts of the product accumulation curves (up to 10 min, dependent on the reaction rate), excluding the initial region, where mixing the reagents and solubilization of the preparation affected the optical density.

### Kinetics of the Substrate and Inhibitor Binding

To compare the catalytic rates of the non-homogeneous preparations, their specific activity, i.e., the reaction rate normalized per mg of protein in the assay system, was used as a measure of the reaction rate *v* or its maximal value *V*_*max*_, attained at the substrate saturation, throughout this study. Accordingly, *a* and *A*_*max*_ are used as the equivalents of *v* and *V*_*max*_, in the text.

Non-linear regression was used to approximate the experimental dependences of the reaction rates on the OG or OA concentration according to the Michaelis-Menten equation ($a=\frac{A_{\max}*\left[S\right]}{K_{m}+\left[S\right]}$) or its sum ($a=\frac{A_{\max}*\left[S\right]}{K_{m}+\left[S\right]}+\frac{A_{\max,2}*\left[S\right]}{K_{m,2}+\left[S\right]}$). The former equation describes one type of the active sites. When the simulation by the model with one type of active sites resulted in a poor fitting of the experimental data (*R*^2^ < 0.98), the data were approximated by the model assuming the two independent types of the active sites with different affinities toward 2-oxo substrates (*K*_*m*,1_ < *K*_*m*,2_). The assumption was based on the presence of the two enzymes, OGDH and OADH, in the employed preparations.

The kinetic data were also analyzed by linear regression, using the known methods of the linearization of the Michaelis-Menten equation in the Lineweaver-Burk ($1/a=1/A_{\max}\left(1+\frac{K_{m}}{\left[S\right]}\right)$) and Eadie-Hofstee ($a=A_{\max}-K_{m}\frac{v}{\left[S\right]}$) coordinates. Due to the several orders of magnitude difference in the values of *K*_*m*,1_ and *K*_*m*,2_, the linear regression required separate analysis of the dependencies in the intervals of the low and high concentrations of 2-oxoadipate. The *K*_*m*_ and *A*_*max*_ values obtained in independent experiments using the non-linear and linear regression analyses of the experimental data, were averaged and presented as Mean ± Standard Error of Mean.

To determine the inhibition constants (*K*_*i*_), the effective values of $K_{m}^{eff}$ and $A_{\max}^{eff}$, calculated from the hyperbolic or linear approximations as described above, were plotted against the concentration of the inhibitors ([I]). The linear regression of the equation $K_{m}^{eff}/A_{\max}^{eff}=K_{m}/A_{\max}\left(1+\frac{\left[I\right]}{K_{i}}\right)$, (Cornish-Bowden, 1979) was applied to find the Intercept (*K*_*m*_/*A*_max_) and Slope (*K*_*m*_/(*A*_max_*K_*i*_)) of a dependence. The values of *K*_*i*_ were determined as the ratio of the corresponding Intercepts to the Slopes. In the two-site model, the $K_{i}^{\left(1\right)}$ and $K_{i}^{\left(2\right)}$ were calculated separately from the linear approximations of $K_{m,1}^{eff}/A_{max,1}^{eff}$ ***vs*. [I]** and $K_{m,2}^{eff}/A_{max,2}^{eff}$ ***vs*. [I]** dependences, respectively. The standard errors of *K*_*i*_ were determined from means and standard errors of the Intercepts and the Slopes, using the previously described formula for standard deviation of a fraction (Bunik et al., 1999). The *K*_*i*_ values obtained from the non-linear and linear regression analyses of the experimental data were averaged and presented as Mean ± Standard Error of Mean.

### Structural Analysis

Sequences of rat OGDH, rat OGDHL, rat OADH, human OADH and OGDH from *Mycobacterium smegmatis* were downloaded from the UniprotKB database (www.uniprot.org, Uniprot, 2019) under the accession numbers Q5XI78, D3ZQD3, Q4KLP0, Q96HY7, and A0R2B1, respectively. The sequences were aligned in JalView version 2.11 (Waterhouse et al., 2009), using the built-in Muscle algorithm (Edgar, 2004) with default parameters. N-terminal mitochondrial targeting sequences were identified by TargetP-2.0 web-server (Almagro Armenteros et al., 2019).

Structure of rat OGDH was modeled using SWISSModel web-server (Waterhouse et al., 2018), with OGDH from *M. smegmatis* in complex with the post-decarboxylation ThDP adduct [PDB ID: 2Y0P, (Wagner et al., 2011)] serving as a template. The modeled structure was superimposed onto that of human DHTKD1 [PDB ID: 6SY1, (Bezerra et al., 2020)]. Each of the structures was then aligned to *M. smegmatis* OGDH in complex with SP^*^ThDP (PDB ID: 6R29, [Wagner et al., 2019)]. Then, SP in its adduct with ThDP was substituted by GP or AP. In each of the phosphonate^*^ThDP adducts, the phosphonate moieties were positioned in the same orientation as SP^*^ThDP in *M. smegmatis* OGDH, and stereochemical restraints dictionaries, generated with the Jligand software (Lebedev et al., 2012), were applied. All superimpositions and image renderings were done in PyMol 2.4 (Schrödinger, LLC).

### Analysis of the Stability of Synthesized Phosphonates During Storage

Stability of the purified phosphonates and their esterified precursor was analyzed by NMR after storage under the inert (pure argon) or ambient (air) atmosphere and in water solutions. ^1^H, ^13^C and ^31^P NMR spectra were recorded at 400, 100.6, and 161.9 MHz, respectively with a Bruker Avance 400 spectrometer. Chemical shifts were calculated using D_2_O or CDCl_3_ as reference standards for trisodium salts and triesters, respectively. The spectra of the phosphonates and their esters before and after storage are shown in Supplementary Figures 1, 2 correspondingly.

### Statistics

Data are presented as Mean ± Standard Error of Mean. Regression analysis was done in GraphPad Prism 8, Fitting the experimental data to a model by non-linear regression was estimated by coefficients of determination (*R*^2^), equivalent to a coefficient of correlation (*r*) used in linear regression.

## Results

### Tissue-Specific Kinetic Parameters of the OGDH and OADH Reactions Correspond to the Isoenzymes Expression

Kinetic analysis of the 2-oxo substrate saturation of the studied enzymes employs the assays of the overall OGDH and OADH reactions, catalyzed by the multienzyme complexes including the 2-oxo acid dehydrogenases as their first components. Purification of these multienzyme complexes to a homogeneous state is hindered by the well-recognized problem of the dissociation of the complex components during the isolation. Specific problem with isolating the homogeneous OADH and OGDH complexes, whose second and third components are the same, is potential formation of the hybrid complex through the exchange of the peripheral subunits, OADH and OGDH. Obviously, such an exchange should be promoted in the concentrated and/or detergent-treated fractions of the two complexes during their isolation. On the other hand, our recent work has shown that OADH in mammalian tissues is significantly modified, compared to the recombinant enzyme (Boyko et al., 2020), which limits the biological significance of the studies of the OADH regulation using the recombinant enzyme. Hence, the tissue fractions with the complexes enriched to a degree allowing for a robust detection of the enzymatic activities, have been used in the current study.

In order to differentiate the contributions of OADH and OGDH to the reactions with OA and OG in such preparations, the enzyme complexes from the rat tissues with different expression of the isoenzymes are used. According to the transcriptomics data (Artiukhov et al., 2020), the rat heart possesses mainly OGDH (*DHTKD1*/*OGDH* mRNA ratio ≈ 0.01), while the liver contains comparable levels of the two isoenzymes (*DHTKD1*/*OGDH* ≈ 1.6). In view of the similar substrate specificity of *OGDH* and another, the *OGDHL*-encoded isoenzymes (Bunik et al., 2008), added by a minor content of the *OGDHL* transcripts (≤ 1% of *OGDH*) in either the heart or liver (Bunik et al., 2008; Artiukhov et al., 2020), the kinetic contribution of the *OGDHL*-encoded OGDH-like isoenzyme in these tissues is neglected.

**Figure 2:**
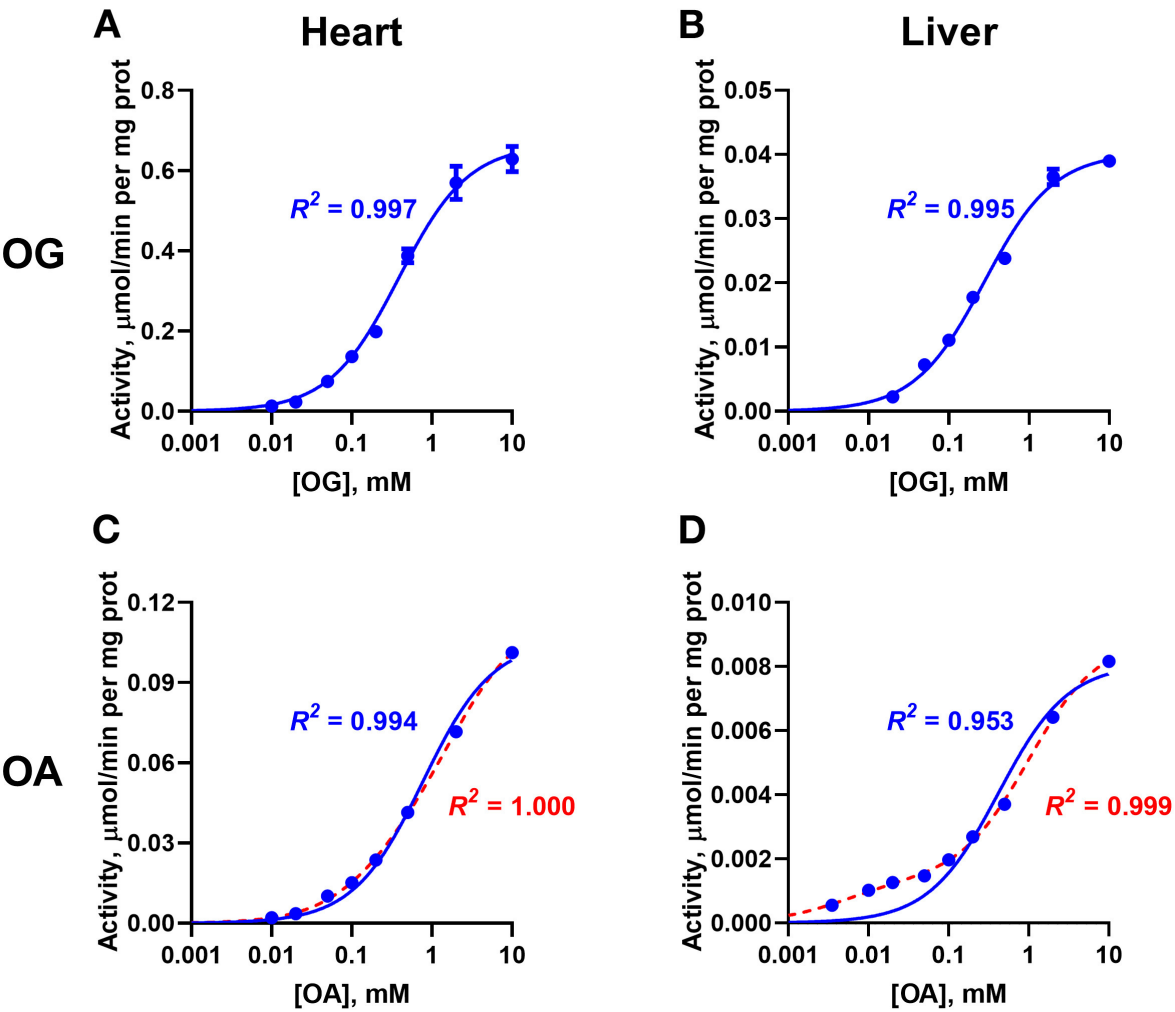
Comparative kinetic analysis of the 2-oxo substrate concentration dependencies of the OGDH and OADH reactions, catalyzed by the enzyme preparations from the rat heart and liver. The reactions were started by the addition of the complexes-enriched enzyme preparations from the rat heart **(A,C)** or liver **(B,D)**, to the assay medium. The dependences of the specific activities of the preparations on the concentrations of 2-oxoglutarate (OG, **A,B**) or 2-oxoadipate (OA, **C,D**) are shown. Experimental data are fitted by the Michaelis-Menten equation (blue lines) or by a sum of the two Michaelis-Menten equations (red lines), with the coefficients of determination (*R*^2^) indicated in the corresponding color. The substrate saturation parameters are presented in Table 1.

Analysis of the dependences of the enzymatic activities from the rat heart and liver on the concentration of OG and OA are shown in Figure 2. The kinetic analysis demonstrates that saturation with OG in both the heart and liver preparations is well fitted by the one-site model (*R*^2^ > 0.99, Figures 2A,B). This finding indicates that the OADH isoenzyme, which is abundant in the liver, does not contribute significantly to the OGDH reaction even in the tissue with a comparable expression of both isoenzymes. Alternatively, the data would agree with similar affinities of OGDH and OADH to OG, but the study with the complexes constructed from recombinant component does not support this assumption (Nemeria et al., 2017, 2018). Contrary to the oxidative decarboxylation of OG, that of OA demonstrates the different kinetics for the two tissues. For the enzyme preparation from heart, saturation with OA is also well-approximated by a Michaelis-Menten model (*R*^2^ = 0.995, blue line in Figure 2C), in accordance with the abundance of the OGDH isoenzyme only. However, in the OADH reaction catalyzed by the enzyme preparation from liver, the dependence of activity on the OA concentration does not conform to the standard Michaelis-Menten approximation. The poor fitting of the experimental data with such a model (*R*^2^ = 0.953, blue line in Figure 2D) is due to the systemic deviation of the experimental dependence from the simulated one in the region of low OA concentrations (Figure 2D). Observation of this deviation only with the liver preparation where OADH is abundant (Figures 2C,D), indicates specific contribution of a *DHTKD1*-encoded isoenzyme to the OADH reaction, which is kinetically different from that of the *OGDH*-encoded isoenzyme. Accordingly, the experimental data has been fitted by a model, assuming two types of independent binding sites. This model, described by a sum of two Michaelis-Menten equations, results in a much better fit to the experimental data (*R*^2^ = 0.999, red line in Figure 2D), than the model assuming the existence of a single type of binding sites (*R*^2^ = 0.953, blue line in Figure 2D).

The kinetic parameters of the reactions with the two substrates, OG and OA, characteristic of the substrate binding sites present in the cardiac and hepatic preparations, are shown in Table 1. The two preparations show similar values of $K_{m}^{OG}$, i.e., 0.45 ± 0.06 and 0.27 ± 0.06 mM (Table 1). However, in the OGDH reaction, maximal specific activity of the cardiac preparation is much higher (0.73 ± 0.05 μmol/min per mg protein) than that of the liver preparation (0.044 ± 0.005 μmol/min per mg protein) (Table 1). Such difference in the activity agrees well with an order of magnitude higher *OGDH* expression in the heart vs. liver, both at the mRNA (5.5-times higher) and protein (10.5-times higher) levels (Artiukhov et al., 2020).

**Table 1:** Kinetic parameters of the dependencies of the OGDH and OADH reactions catalyzed by the complexes-enriched preparations from the rat heart and liver, on concentrations of the 2-oxo substrates.

**Parameters**	**OG saturation**		**OA saturation**		
	**Heart**	**Liver**	**Heart**	**Liver**	
				**Low affinity**	**high affinity**
*K_*m*_*, mM	0.45 ± 0.06	0.27 ± 0.06	0.55 ± 0.07	0.45 ± 0.12	0.0077 ± 0.0008
*A_*max*_*, μmol/min per mg of protein	0.73 ± 0.05	0.044 ± 0.005	0.102 ± 0.008	0.0075 ± 0.0002	0.0018 ± 0.0001

*Michaelis-Menten equation is used for analysis of the OG saturations presented in Figures 2A–C. A sum of two Michaelis-Menten equations characterizing the low-affinity and high-affinity binding sites for OA, is used for analysis of the OA saturation presented in Figure 2D. The mean values of parameters are calculated via averaging the results of non-linear and linear regression analyses as described in Methods*.

The saturation of the cardiac enzyme preparation with OA gives the $K_{m}^{OA}$ of 0.55 ± 0.07 mM (Table 1). This value is close to that known for the recombinant OGDH complex catalyzing oxidative decarboxylation of OA as its alternative substrate (0.52 mM, Nemeria et al., 2017). A 7-fold ratio of the maximal specific activity of the cardiac enzyme preparation under the saturation with OG (0.73 ± 0.05 μmol/min per mg protein), compared to that with OA (0.102 ± 0.008 μmol/min per mg protein), is also in good agreement with a 5-fold ratio of $K_{cat}^{OG}$/$k_{cat}^{OA}$, observed for the recombinant OGDH complex (Nemeria et al., 2017).

The biphasic saturation with OA in the hepatic enzyme preparation reveals the two types of active sites with $K_{m,1}^{OA}$ = 0.008 ± 0.001 mM and $K_{m,2}^{OA}$ = 0.45 ± 0.12 mM (Table 1). A 60-fold difference in the affinities of the two types of the sites in the hepatic enzyme preparation is higher than a 35-fold difference revealed for the recombinant human OADH ($K_{m}^{OA}$ = 0.015 mM) and OGDH ($K_{m}^{OA}$ = 0.52 mM) (Nemeria et al., 2017, 2018), indicating a better ability of the native OADH complex to discriminate the cognate substrate, compared to that of the recombinant OADH complex. The specific activities of the high- and low-affinity OA-binding sites of the hepatic preparation in the OADH reaction at the saturation with OA correspond to 0.0075 ± 0.0002 and 0.0018 ± 0.0001 μmol/min per mg protein (Table 1), respectively. Assuming comparable abundances of the *OGDH-* and *DHTKD1-*encoded mRNAs and proteins in the liver (Artiukhov et al., 2020) and a 2-fold higher $k_{cat}^{OA}$ of recombinant OADH complex (6.0 s^−1^), compared to $k_{cat}^{OA}$ of recombinant OGDH complex (2.7 s^−1^) (Nemeria et al., 2017, 2018), a higher contribution of the high-affinity vs. low-affinity sites to the OADH reaction, catalyzed by the hepatic preparation, can be expected. However, the maximal specific activity of the high-affinity sites determined in the assay is only 25% of that of the low-affinity sites. This relatively low contribution corresponds well to a more successful competition of OGDH with OADH for the common second component of the complex, suggested earlier from the comparison of the activities of the OADH complex in different rat tissues (Artiukhov et al., 2020). Besides, it supports our recent finding (Boyko et al., 2020) that significant part of OADH expressed in the liver may actually exist as an isolated enzyme, catalyzing non-oxidative decarboxylation of OA, which does not require formation of the multienzyme complex for the oxidative decarboxylation of OA, studied in this work.

Thus, the kinetic analysis of the OGDH and OADH reactions catalyzed by the complexes-enriched preparations from the rat heart and liver provides the estimations of the relative affinities of the complexes, existing in the mammalian tissues, to their alternative substrates OG and OA. The determined kinetic parameters also suggest that only a small part of total OADH synthesized in the liver contributes to the overall complex-catalyzed reaction of oxidative decarboxylation of OA.

### Kinetic Analysis of the Inhibition of the OGDH and OADH Reactions by the Phosphonate Analogs of 2-oxo dicarboxylates

**Figure 3:**
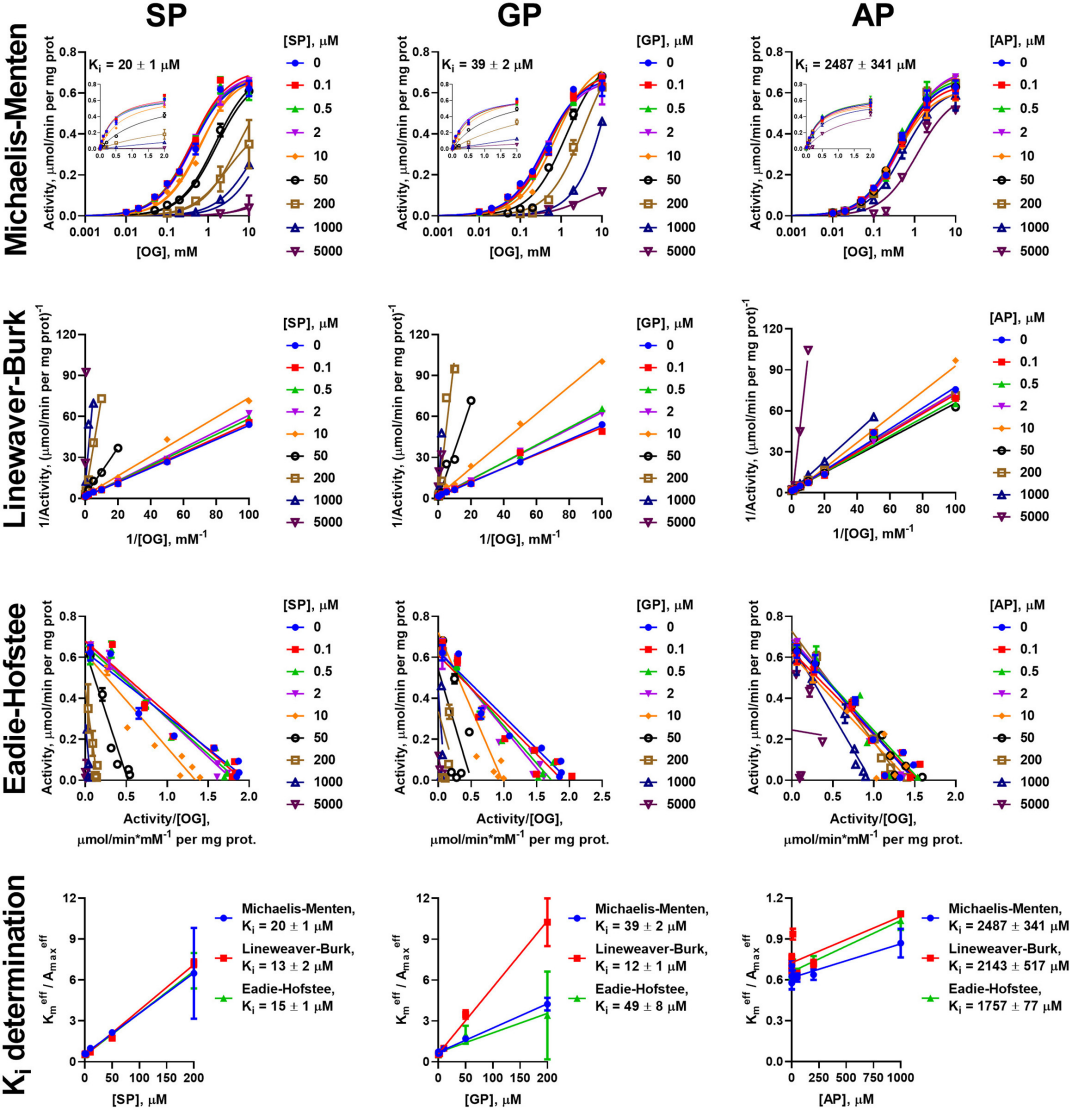
Kinetic analysis of inhibition of OGDH reaction, catalyzed by the enzyme preparation from the rat heart, by the phosphonate analogs of 2-oxo acid substrates. Plots in semi-log coordinates show the dependence of specific activity on 2-oxoglutarate (OG) concentration (0–10 mM, log scale) at different concentrations of SP, GP, or AP, fitted by Michaelis-Menten equations. Inset graphs show the same dependences using linear scale, cropped at 2 mM. Approximation of the experimental data by linear regression in the Lineweaver-Burk and Eadie-Hofstee coordinates and determination of *K*_*i*_ values using linear dependencies of $K_{m}^{eff}/A_{max}^{eff}$ on [I] are shown below the non-linear regression. The *K*_*i*_ values obtained in the three types of coordinates are presented in Table 2.

**Figure 4:**
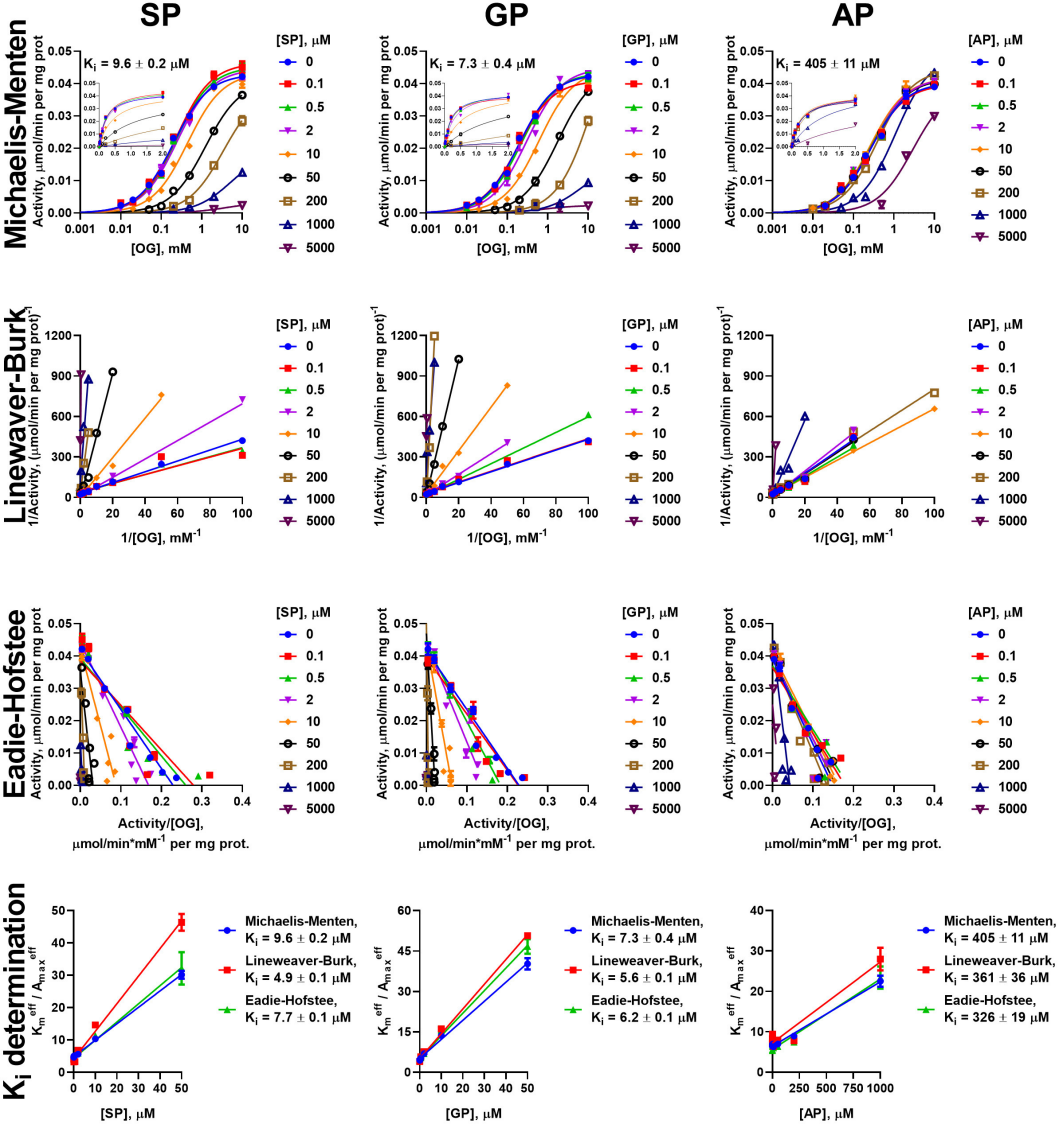
Kinetic analysis of inhibition of OGDH reaction, catalyzed by the enzyme preparation from liver, by the phosphonate analogs of 2-oxo acid substrates. The assay conditions and graph layout are the same as in Figure 3, except the enzyme preparation from the rat liver is used. *K*_*i*_ values are presented in Table 2.

**Figure 5:**
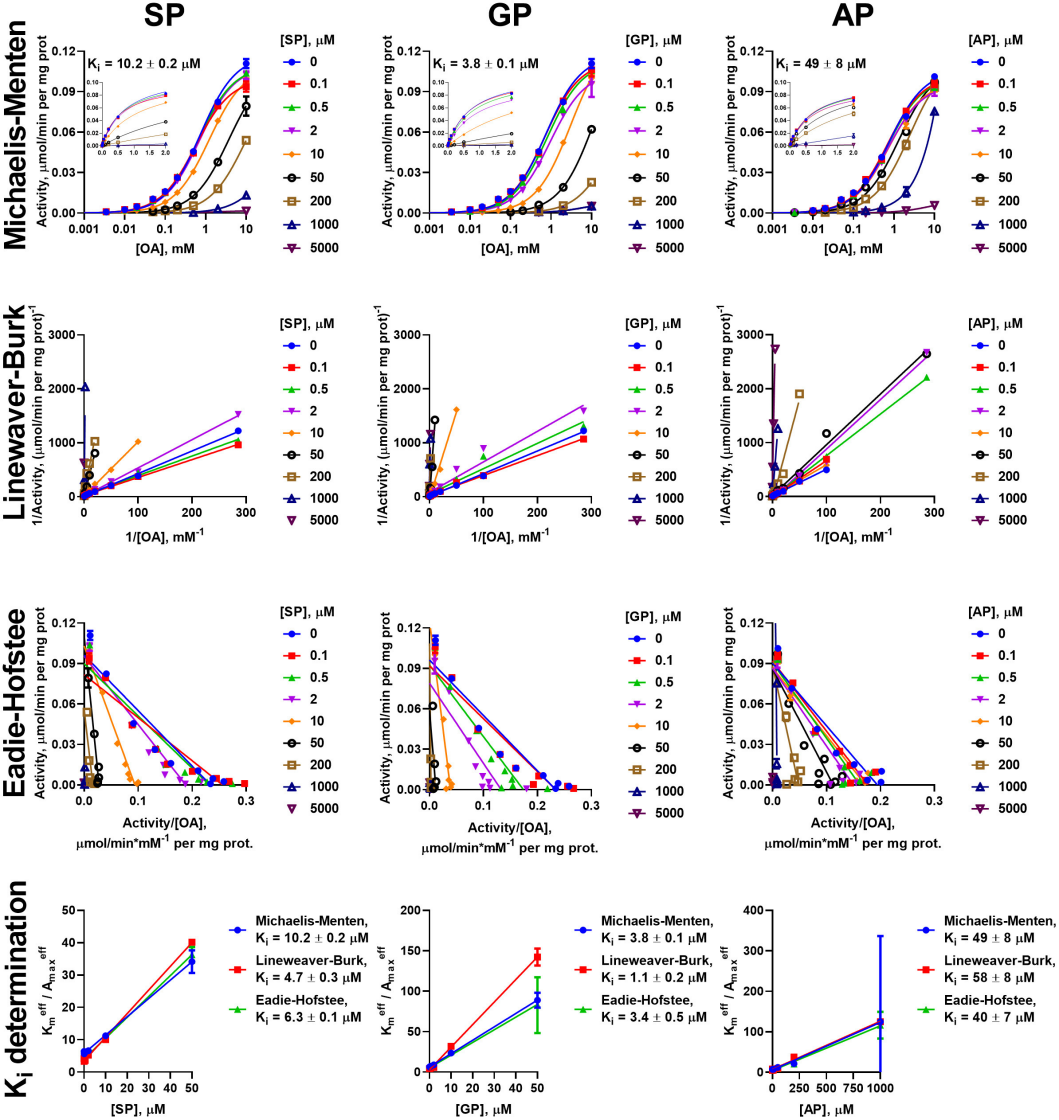
Kinetic analysis of inhibition of OADH reaction, catalyzed by the enzyme preparation from the rat heart, by the phosphonate analogs of 2-oxo acid substrates. The assay conditions and graph layout are the same as in Figure 3, except 2-oxoadipate (OA) is used instead of 2-oxoglutarate. *K*_*i*_ values are presented in Table 3.

Similar to the analysis of the enzyme saturations with the 2-oxo substrates, analysis of the data on the inhibition of the oxidative decarboxylation of OG and OA by their phosphonate analogs requires using different models, dependent on the 2-oxo substrate and enzyme preparation. Experimental data on the OG saturation of the enzyme preparations from both heart and liver in presence of inhibitors (SP, GP, and AP) are satisfactorily approximated (*R*^2^ > 0.98) by the Michaelis-Menten equation (Figures 3, 4). The same holds for the OA saturation of the cardiac enzyme preparation possessing one type of the OA-binding sites (Figure 5).

**Figure 6:**
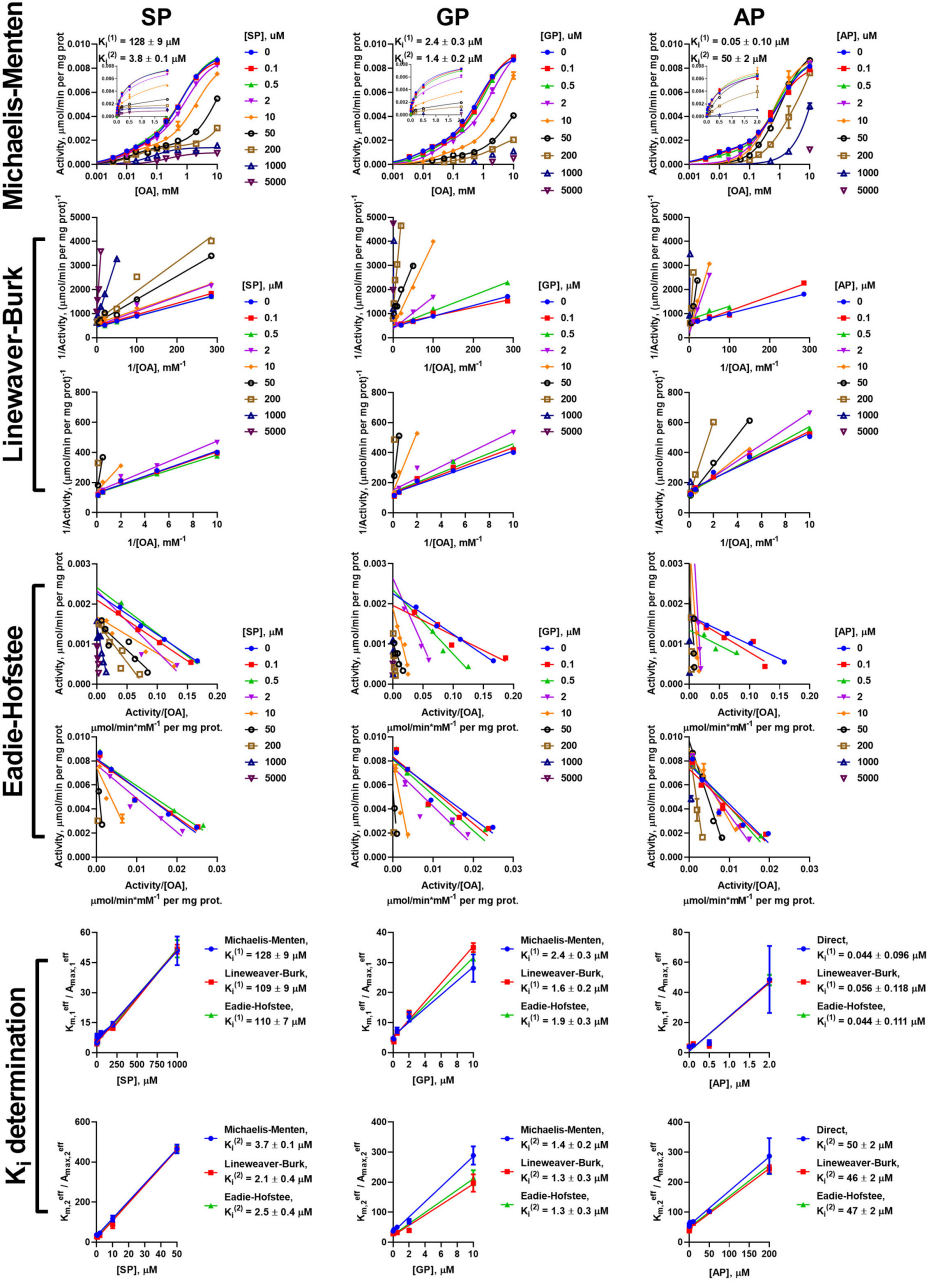
Kinetic analysis of inhibition of OADH reaction, catalyzed by the enzyme preparation from the liver, by the phosphonate analogs of 2-oxo acid substrates. The assay conditions are the same as in Figure 5. Approximation of the experimental data by a sum of two Michaelis-Menten equations is shown in the semi-log coordinates at different concentrations of SP, GP, or AP. Inset graphs show the same dependences using linear scale, cropped at 2 mM. The linear regression of experimental data in the Lineweaver-Burk and Eadie-Hofstee coordinates shows the separate analysis of the inhibition in the different ranges of 2-oxoadipate (OA) concentrations. *K*_*i*_ values obtained in the three types of coordinates for the sites saturated by high ($K_{i}^{\left(1\right)}$) and low ($K_{i}^{\left(2\right)}$) OA concentrations are given in Table 3.

However, the two types of the OA-binding sites in the enzyme preparation from liver (Figure 2D and Table 1), are distinguished also by their different reactivities toward the phosphonates. In this case, when the approximation by the Michaelis-Menten equation of experimental data on OA saturation without the inhibitors is not satisfactory (*R*^2^ < 0.98), the *R*^2^ values characterizing the fitting of the experimental data to the model vary, depending on the phosphonate used and its concentration. For instance, at a fixed concentration of 0.05 mM, *R*^2^ decreases to 0.822 at 50 μM SP, remains unchanged with GP (*R*^2^ = 0.930) and improves with AP (*R*^2^ = 0.998). This finding indicates that the preferential inhibition of the low-affinity sites by SP increases the relative contribution of the high-affinity sites to the OADH reaction, thus reducing *R*^2^ for the one-site model. In contrast, preferential inhibition by AP of the high-affinity OA-binding sites decreases their contribution to the catalysis, leading to the improved fitting of the experimental data in the presence of AP to the one-site model. No changes in *R*^2^ suggest similar reactivity of the different sites to GP. Thus, in general, the substrate saturation curves in the presence of the increasing concentrations of SP, GP and AP have been approximated by a sum of two Michaelis-Menten equations. Considering the difference in $K_{m,1}^{OA}$ and $K_{m,2}^{OA}$ of two orders of magnitude (Table 1), the inhibition has been assessed separately in a low (0–0.05 mM) and a high (0.1–10 mM) interval of OA concentrations (Figure 6).

To investigate the mechanism of inhibition, changes in the effective kinetic parameters ($K_{m}^{eff}$ and $A_{max}^{eff}$) at different concentrations of the inhibitors are analyzed. For the OADH and OGDH reactions, catalyzed by the cardiac or hepatic enzyme preparations, increasing the inhibitor concentration up to 200 μM primarily decreases the affinity to the 2-oxo substrate without significant changes in the maximal specific activity (Supplementary Table 1). This indicates that the primary mechanism of the SP, GP, and AP inhibition of the OGDH and OADH reactions under the studied conditions corresponds to competitive inhibition. However, at high concentrations of the phosphonates also decreases in $A_{max}^{eff}$ could be observed, although more pronounced with SP than with GP or AP. This agrees well with the ability of the phosphonates to form tight, slow dissociating complexes with the active sites of 2-oxo acid dehydrogenases (reviewed in Bunik et al., 2013; Artiukhov et al., 2016).

Due to the prevalence of the competitive inhibitory component over non-competitive one, the linear dependences of ratios of effective *K*_*m*_ to maximal specific activity on the concentration of inhibitors was utilized to calculate the *K*_*i*_ values, as shown in Cornish-Bowden (1979). To ensure consistency, the effective kinetic parameters were determined not only from hyperbolic approximations by Michaelis equation, but also by linear approximations in Lineweaver-Burk and Eadie-Hofstee coordinates (Figures 3–6). These linear transformations also confirm the competitive nature of the inhibition as the approximations of 2-oxo substrate saturation at different concentrations of the phosphonates intersect at *y*-axes (Figures 3–6). Catalysis of the OGDH reaction by the enzyme preparations from heart or liver is affected similarly by the tested phosphonates: SP (*K*_*i*_ = 16 ± 2 μM and 7.4 ± 1.4 μM for heart and liver, respectively) and GP (*K*_*i*_ = 33 ± 11 μM and 6.3 ± 0.5 μM for heart and liver, respectively) demonstrate *K*_*i*_ values in micromolar range, whereas AP (*K*_*i*_ = 2,129 ± 211 μM and 364 ± 23 μM for heart and liver, respectively) is about two orders of magnitude less effective (Table 2). Similarly, OADH reaction catalyzed by cardiac preparation is inhibited by SP (*K*_*i*_ = 7.1 ± 1.6 μM) and GP (*K*_*i*_ = 2.8 ± 0.8 μM) more efficiently than by AP (*K*_*i*_ = 49 ± 5 μM) (Table 3). Thus, the relative power of SP, GP, and AP in inhibiting the enzyme preparation from heart is similar for the OGDH and OADH reactions, as it is catalyzed by the same isoenzyme (OGDH).

**Table 2:** Kinetic parameters of the inhibition of OGDH reaction catalyzed by the enzyme preparations from the rat heart or liver.

**Parameters**	**Heart**				**Liver**			
	**MM**	**LB**	**EH**	**Average**	**MM**	**LB**	**EH**	**Average**
$K_{i}^{SP}$, μM	20 ± 1	13 ± 2	15.5 ± 1	16 ± 2	9.6 ± 0.2	4.9 ± 0.1	7.7 ± 0.1	7.4 ± 1.4
$K_{i}^{GP}$, μM	39 ± 2	12 ± 1	49 ± 8	33 ± 11	7.3 ± 0.4	5.6 ± 0.1	6.2 ± 0.1	6.3 ± 0.5
$K_{i}^{AP}$, μM	2487 ± 341	2143 ± 517	1757 ± 77	2129 ± 211	405 ± 11	361 ± 36	326 ± 19	364 ± 23

*The parameters of the 2-oxoglutarate saturation in the presence of inhibitors are obtained from approximations of the experimental data by Michaelis-Menten equations (MM) or their linearization in the Lineweaver-Burk (LB) or Eadie-Hofstee (EH) coordinates. K_i_ values are calculated from the linear dependencies of $K_{m}^{eff}/A_{max}^{eff}$ on [I] (Figures 3, 4)*.

**Table 3:** Kinetic parameters of the inhibition of OADH reaction catalyzed by the enzyme preparations from the rat heart or liver.

**Parameters**	**Heart**				**Liver, high [OA]**				**Liver, low [OA]**			
	**MM**	**LB**	**EH**	**Average**	**MM**	**LB**	**EH**	**Average**	**MM**	**LB**	**EH**	**Average**
$K_{i}^{SP}$, μM	10.2 ± 0.2	4.7 ± 0.3	6.3 ± 0.1	7.1 ± 1.6	3.7 ±0.1	2.1 ± 0.4	2.5 ±0.4	2.8 ± 0.5	128 ± 9	109 ± 9	110 ± 7	116 ± 6
$K_{i}^{GP}$, μM	3.8 ± 0.1	1.1 ± 0.2	3.4 ± 0.5	2.8 ± 0.8	1.4 ±0.2	1.3 ± 0.3	1.3 ± 0.02	1.3 ± 0.2	2.4 ± 0.3	1.6 ± 0.2	1.9 ± 0.3	2.0 ± 0.2
$K_{i}^{AP}$, μM	49 ± 8	58 ± 8	40 ± 7	49 ± 5	50 ± 2	46 ± 2	47 ± 2	47 ± 1	0.044, 0.096	0.056 ± 0.118	0.044 ± 0.111	0.048 ± 0.004

*The parameters of the 2-oxoadipate (OA) saturation in the presence of inhibitors are obtained from approximations of the experimental data by Michaelis-Menten equations (MM) and their linearization in the Lineweaver-Burk (LB) or Eadie-Hofstee (EH) coordinates. For the cardiac enzyme preparation, the whole range of OA concentrations (0–10 mM) is analyzed (Figure 5). For the enzyme preparation from liver, the data points with high (0.1–10 mM) and low (0–0.05 mM) OA concentrations are analyzed separately to determine the kinetic parameters for the sites with the low and high affinities toward OA, respectively (Figure 6). K_i_ values are calculated from the linear dependencies of $K_{m}^{eff}/A_{max}^{eff}$ on [I] (Figures 5, 6)*.

The data on the SP, GP, and AP inhibition in the different ranges of OA concentrations in the hepatic preparation were analyzed separately at the low (0–0.05 mM) and high (0.1–10 mM) ranges of OA concentrations. At 0.1–10 mM 2-oxoadipate, the inhibitory pattern of OADH reaction in hepatic preparation is similar to that of cardiac preparation: *K*_*i*_ values for SP (2.8 ± 0.5 μM) and GP (1.3 ± 0.02 μM) are more than an order of magnitude lower than that for AP (47 ± 1 μM). However, up to 0.05 mM 2-oxoadipate, the opposite inhibitory pattern is observed: the lowest *K*_*i*_ value is for AP (0.048 ± 0.004 μM), followed by those for GP (2.0 ± 0.2 μM) and SP (116 ± 6 μM). This finding corroborates the existence of two independent types of the active sites, binding OA, and phosphonates. Compared to the high-affinity OA-binding sites, more sensitive to AP, those with the low affinity to OA have a higher affinity to SP. As shown above, the former sites correspond to OADH catalysis by the *DHTKD1-*encoded protein (OADH), whereas the latter—by the *OGDH(L)*-encoded protein (OGDH). GP binding at both types of the active sites does not differ as significantly as the binding of SP or AP.

The presence of the two types of independent OA-binding sites in the hepatic preparation results in an interesting inhibition pattern, when the competitive action of SP vs. OA in each of the sites (Figure 6 and Supplementary Table 1) is accompanied by a stronger inhibition of the OADH reaction by SP at higher, than lower, OA concentrations (Figure 6). Although this pattern is normally not inherent in the competitive inhibition, it confirms the two types of independent OA binding sites, where the sites with low-affinity to OA have a higher reactivity to SP, than the sites with the high affinity to OA.

### Comparative Analysis of the OGDH and OADH Structures Elucidates the Features Favoring the Binding of Bulkier Ligands to OADH

**Figure 7:**
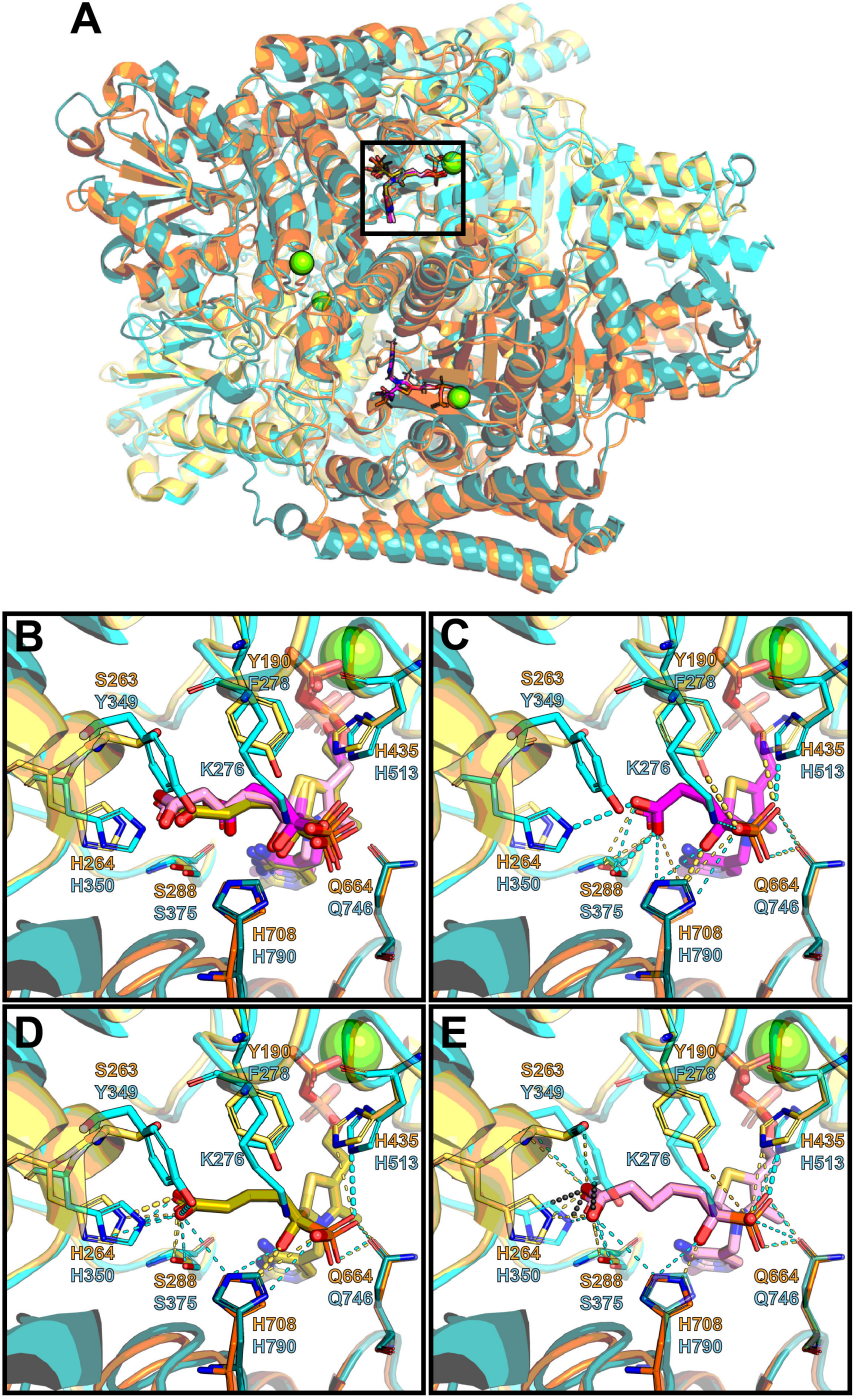
Structural analysis of the OGDH and OADH complexes with ThDP and phosphonate analogs of the 2-oxo substrates. Modeled structure of rat OGDH (chain A: cyan; chain B: teal; cartoon representation with protein side chains not shown) is superimposed onto human OADH (PDB ID: 6SY1; chain A: yellow; chain B: orange; cartoon representation with protein side chains not shown) and to OGDH from *Mycobacterium smegmatis* in complex with SP*ThDP (PDB ID: 6R29; SP*ThDP: magenta; Mg^2+^: green; protein not shown). SP*ThDP is further substituted by GP*ThDP (olive) and AP*ThDP (pink)in the same conformation, applying stereochemical restraints (see Methods). **(A)** General overview of the OGDH and OADH superimposition, showing localization of the phosphonate*ThDP adducts at the subunit interface. **(B–E)** Zoomed view of the active site pocket, marked by rectangle in **(A)**, with side chains involved in phosphonate binding are shown as sticks, and labeled for the superimposed adducts **(B)** and for the SP*ThDP **(C)**, GP*ThDP **(D)**, or AP*ThDP **(E)** adducts separately. Polar contacts (< 4.2Å) between the phosphonates and OGDH or OADH residues are shown as cyan and yellow dashes respectively; thicker lines correspond to favorable orientation of two atoms and optimal distance between them for a hydrogen bond formation. Gray dotted lines in **(E)** correspond to predicted steric clashes (< 2.1Å) between AP and OGDH residues.

Since no structure of mammalian OGDH is currently available, a model of rat OGDH was obtained via homology modeling, using as a template the structure of *M. smegmatis* OGDH in complex with the post-decarboxylation ThDP adduct formed after addition of OG [PDB ID: 2Y0P (Wagner et al., 2011)]. Superposition of the rat OGDH model to the coordinates of OGDH from *M. smegmatis* in complex with SP (PDB ID: 6R29) and substitution of SP in this complex by GP and AP, as described in Methods, allows one to assess potential interactions of the phosphonate ligands with the active site of the rat OGDH (Figure 7A). As shown in the *M. smegmatis* enzyme (Wagner et al., 2019), SP is covalently bound to ThDP forming a pre-decarboxylation complex mimic. Substitution of SP in this complex with GP or AP, applying appropriate stereochemical restraints and keeping the same orientation of the acyl chain as in SP, is shown in Figure 7B. In these complexes, SP is not so close to the OGDH Tyr349 residue (Figure 7C), as GP (Figure 7D). However, the additional, compared to SP, interaction of GP with the hydroxyl group of Tyr349 is not manifested in a better binding of GP vs. SP in kinetics experiments (Table 2). Likely, the interaction of GP with Tyr349 reorients the phosphonate carboxyl group, weakening the GP interactions with His350 and Ser375, compared to those with SP (Figures 7C,D), thus explaining the equal binding of SP and GP to OGDH (Table 2). In contrast, substitution of the SP adduct with AP (Figure 7E) reveals steric clashes with the phenyl group of Tyr349. This structural feature is consistent with the much lower affinity of OGDH to AP than to SP and GP (Table 2).

**Figure 8:**
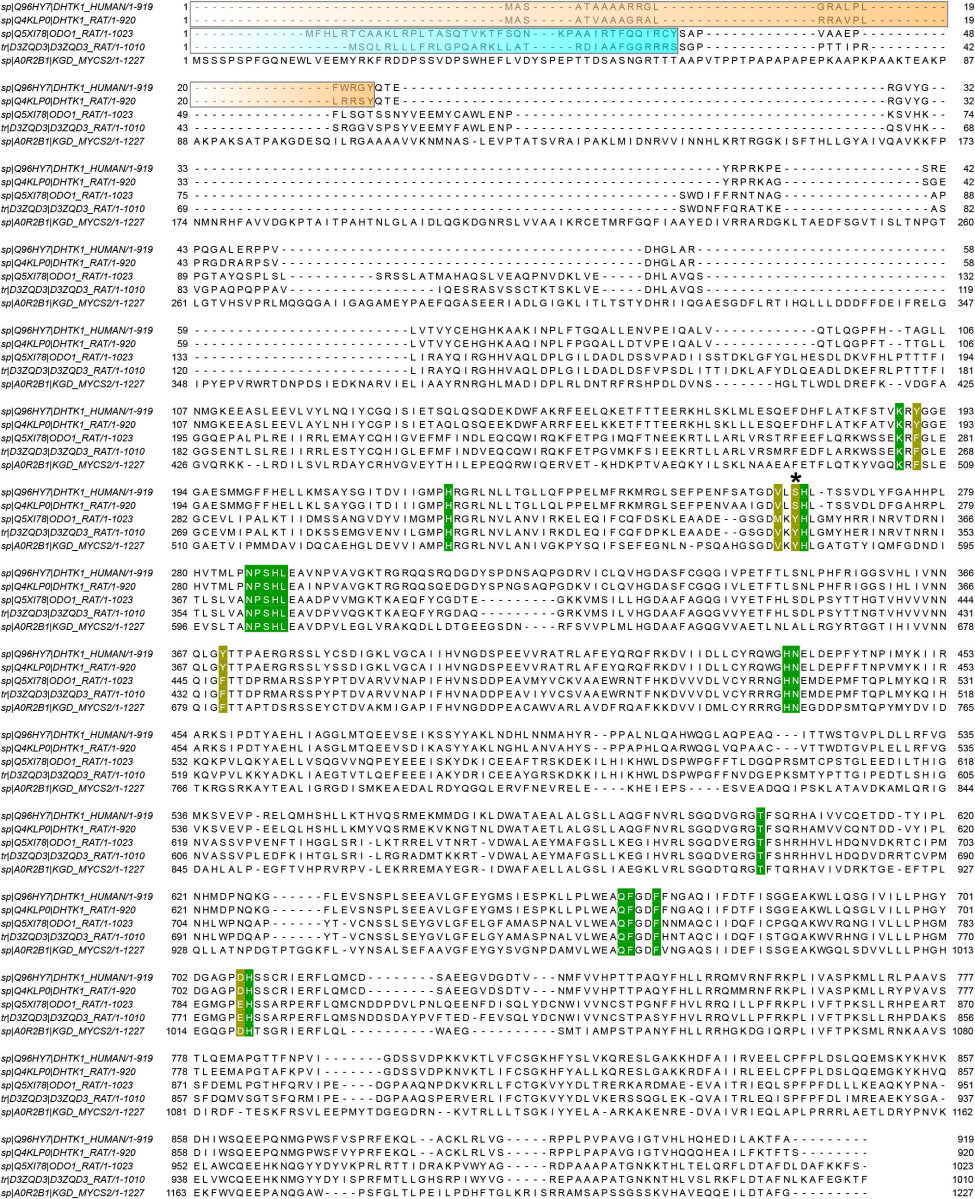
Alignment of the OADH and OGDH isoenzymes. Sequences of human OADH (DHTK1_HUMAN), rat OADH (DHTK1_RAT), rat OGDH (ODO1_RAT), rat OGDHL (D3ZQD3_RAT) and OGDH from *Mycobacterium smegmatis* (KGD_MYCS2) are aligned in JalView 2.11. Orange and cyan blocks indicate mitochondrial targeting sequence in mammalian OADH and OGDH sequences, respectively, predicted by TargetP-2.0. Residues located within 4.6 Å from adipoyl phosphonate (AP) moiety in human OADH structure with its ThDP ligand replaced by the phosphonate*ThDP adducts (see Figure 7) are colored. Those which are the same in all the five structures, are marked in green. The residues which differ in the analyzed sequences, are shown in yellow. The Ser/Tyr residue presumed to be important for the substrate/inhibitor specificity (see Figure 7) is marked by asterisk.

To assess how the phosphonates interact with the active site of OADH, the recently published structure of human OADH was used [PDB ID: 6SY1 (Bezerra et al., 2020)]. The human enzyme has 89% sequence identity with the mature rat protein, i.e., the protein without the N-terminal mitochondrial localization signal, absent in the crystal structure (Figure 8). Hence, the human OADH structure was further used to estimate the interactions of mammalian OADH with the phosphonates. Superimposition of the OADH structure onto the mycobacterial OGDH in complex with SP, followed by the manual replacement of SP by GP or AP, reveals that the carboxyl group of a longer inhibitor (AP) forms multiple polar contacts with side chains of the OADH residues, including Ser263, His264, and Ser288 (Figure 7E). The GP carboxyl group does not interact with Ser263, but shows optimal H-bonding distance to the imidazole group of His264 (Figure 7D), whereas less favorable interactions of the phosphonate carboxyl with Ser288 are suggested in the case of SP (Figure 7C). This is in good accord with kinetics data, showing the strongest OADH inhibition by AP, and the weakest—by SP (Table 3).

Although a number of residues in the active site environment could reorient their side chains upon the ligand binding, which is an interesting area for further studies, the observed differences of the considered OGDH and OADH complexes with the three phosphonates well-agree with the lower efficiency of SP binding to OADH vs. OGDH, with the opposite behavior shown by AP and the similar inhibition properties shown by GP on both enzymes (Table 3). Indeed, similar protein-ligand interactions are observed in both protein complexes with GP (Figure 7D), agreeing with no preferential binding of GP to either the OGDH or OADH active sites, identified in the kinetic experiments (Table 3). Moreover, the enzyme-GP (C6 analog) interactions in the complex presented in Figure 7D well-agree with those observed in the post-decarboxylation complex of mycobacterial OGDH arising after addition of OA (C6 2-oxo acid) [PDB ID: 3ZHU (Wagner et al., 2014)]. Regarding SP, whose carboxyl group forms polar contacts with His350 and Lys276 in OGDH (Wagner et al., 2019), the less efficient binding to OADH agrees with these interactions to appear less favorable (Figure 7C). Furthermore, as noted above, the carboxyl group of a longer inhibitor (AP) may form a hydrogen bond with Ser263 residue of OADH, through its side chain hydroxyl and/or the carbonyl oxygen (Figure 7E). In contrast to OADH, both mammalian and mycobacterial OGDH, as well as the OG-specific mammalian isoenzyme encoded by the *OGDHL* gene, all possess a tyrosine residue at the equivalent positions (Figure 8), likely preventing the effective binding of bulkier ligands (Figure 7E). Among other residues, which are located within 4.6 Å of the phosphonate moieties, most are invariant or show conservative substitutions, such as Phe to Tyr, Val to Met and Glu to Asp, occupying the same space, as seen from the sequences alignment (Figure 8) and structural superposition (Figure 7) of OADH vs. OGDH proteins. Thus, the observed difference between Ser263 in OADH and Tyr349 in OGDH may be a key factor determining specificity in binding of the longer vs. shorter ligands in the enzyme active sites. This substrate specificity determinant has been suggested by our previous sequence analysis (Bunik and Degtyarev, 2008), and the recently published DHTKD1 structure (Bezerra et al., 2020).

### Chemical Stability of the 2-Oxo Phosphonates and Their Esterified Derivatives

In our long-standing work with the phosphonate analogs of dicarboxylic 2-oxo acids (Bunik et al., 1992, 2005; Artiukhov et al., 2020) estimations of their stability upon storage have been accumulated, with the issue being of utmost importance for the reproducibility of biological experiments.

In the form of trisodium salts, SP, GP, and AP demonstrate a high stability upon the shelf storage. As judged from their ^1^H and ^31^P NMR spectra (Supplementary Figure 1), the pure solid forms of the phosphonates may be stored at room temperature for 6 months without any significant destruction (Supplementary Figures 1E,F vs. Supplementary Figures 1C,D and Supplementary Figures 1K,L vs. Supplementary Figures 1H,I). 0.2 M stock solutions of the trisodium salts in water do not degrade after 2 months of storage at −20°C (Supplementary Figure 1G vs. Supplementary Figure 1C and Supplementary Figure 1M vs. Supplementary Figure 1H).

The esterified forms of the phosphonates (Figure 1C) are synthetic precursors of the stable trisodium salts (Bunik et al., 1992, 2005; Artiukhov et al., 2020). In general, the esters of organic acids have an advantage for biological experiments, as they may easily permeate biological membranes due to the absence of charged groups. As a result of de-esterification by intracellular esterases, the *in vivo* inhibition by the esterified phosphonates is observed, although they are inactive as the inhibitors of 2-oxo acid dehydrogenases *in vitro* (Bunik et al., 2005, 2013, 2015; Artiukhov et al., 2016). However, we have observed much lower stability of the esterified phosphonates, compared to the phosphonate salts. At room temperature, pure forms of the 2-oxo phosphonate esters represent oils, which slowly decompose even in argon atmosphere. According to the ^1^H and ^31^P NMR spectra (Supplementary Figure 2), the purity of triethyl esters of SP (TESP) and GP (TEGP) and trimethyl ester of AP (TMAP) is decreased by 4–6% after 6 months of storage under these conditions (Supplementary Figures 2C,D vs. Supplementary Figures 2A,B, Supplementary Figures 2H,I vs. Supplementary Figures 2E,F and Supplementary Figures 2P,Q vs. Supplementary Figures 2M,N). All three esters are highly unstable in air, decomposed almost completely after 5 days of the air exposure. Based on ^1^H and ^31^P NMR spectra (Supplementary Figures 2J,K and Supplementary Figures 2R,S), the decomposition involves the hydrolysis of C-P bond, resulting in the formation of dimethyl or diethyl phosphite, depending on the ester structure, and monoester of residual dicarboxylic acid: monoethyl succinate, monoethyl glutarate and monomethyl adipate for TESP, TEGP, and TMAP, respectively (Supplementary Figure 3). Because the decomposition does not involve the reaction with oxygen, it is not excluded that atmospheric pollution and/or humidity may be responsible for the high decomposition rates in air vs. argon.

The decomposition of the phosphonate esters in aqueous solutions is much slower, compared to the pure compounds exposed to air, and depends on the structure. As shown in Supplementary Figure 2, changes in ^31^P NMR spectra indicate that after 5 days of shelf storage as 0.2 M aqueous solution about 80% TEGP is preserved (Supplementary Figure 2L vs. Supplementary Figure 2E), although TMAP decomposes almost completely (90%) under the same conditions (Supplementary Figure 2T vs. Supplementary Figure 2M).

The investigated stability of the phosphonates (Figure 1B) and their esterified precursors (Figure 1C), which are membrane-permeable pro-inhibitors generating the inhibitors *in vivo*, provides important information for their usage in biological experiments.

## Discussion

In the current work, we have quantified the selectivity of the interaction of the phosphonate analogs of the C5-C7 dicarboxylic 2-oxo acids with the active sites of the isoenzymes encoded by the *OGDH* and *DHTKD1* genes, and revealed structural basis of the selectivity. The findings indicate that AP is a rather specific inhibitor of the *DHTKD1*-encoded OADH, whose interaction with OGDH is much less efficient due to the clashes of AP with an active site tyrosine residue of OGDH, replaced by a serine residue in OADH.

With the presented information on the selective action of SP and AP on OGDH and OADH, correspondingly, the availability of AP opens new ways to decipher physiological role of OADH using the pharmacological regulation of the enzyme. Unlike genetic manipulations of the enzyme expression, which are usually accompanied by compensatory response of the whole metabolic and regulatory networks, the short-term and reversible effects of the enzyme inhibitor may be assessed in specific (patho)physiological states, which is currently required for understanding the significance of OADH function at an organism level. In fact, unlike the well-studied and ubiquitous OGDH, which function is critical for the organism survival, the expression of the *DHTKD1* gene encoding OADH is tissue-specific and depends on a variety of factors. For instance, several types of tumors, including lymphomas and malignant brain tumors, overexpress the *DHTKD1*-encoded OADH (Kiełbus et al., 2015; Papatheodorou et al., 2018), and inhibition of OADH in such tumors by AP may be a plausible therapeutic strategy. The DHTKD1 expression is known to be changed in the age- and metabolic-syndrome-related pathologies, such as obesity, diabetes, neurodegenerations, and inflammatory disorders (Xu et al., 2012, 2018, 2019; Lim et al., 2014; Wu et al., 2014; Kiełbus et al., 2015; Plubell et al., 2018; Sherrill et al., 2018; Timmons et al., 2018; Luan et al., 2020). However, the exact molecular mechanisms underlying the relation between these disorders and OADH function are unknown. Elucidating these mechanisms requires discrimination of the OADH and OGDH activities, which may be of marker significance, in the tissue or cell homogenates (Tsepkova et al., 2017; Artiukhov et al., 2020). Currently, such discrimination is challenging, because ubiquitous OGDH, whose abundance in most of the tissues is much higher than that of OADH (Artiukhov et al., 2020), may catalyze the same reactions *in vitro*, masking the potential OADH contribution. The synthetic analogs of 2-oxo dicarboxylates characterized in this work, may be utilized as tools to solve this problem. As shown above, addition of SP to the assay medium may specifically decrease contribution of the OGDH-catalyzed reaction. For instance, 10^−4^ M SP as a specific OGDH inhibitor with *K*_*i*_ of 10^−5^ M (Table 2) may silence most of OGDH without significantly affecting OADH. Addition of AP may be used to do the same regarding the OADH-catalyzed reaction, as there is an order of magnitude difference in *K*_*i*_ for AP of OGDH and OADH (Table 2). Remarkably, such selectivity in targeting OGDH or OADH is accompanied by no significant interaction of SP or AP with a number of other enzymes transforming organic acids or structurally related compounds (Artiukhov et al., 2020). Our findings justify applications of AP *in vivo* to reveal specific physiological roles of OADH.

## Conclusion

The phosphonate analogs of dicarboxylic 2-oxo acids are quantified as competitive vs. the 2-oxo acid substrates inhibitors of the 2-oxoadipate and 2-oxoglutarate dehydrogenases. Remarkable selectivity of the interactions of succinyl phosphonate with the *OGDH(L)-*encoded 2-oxoglutarate dehydrogenase and adipoyl phosphonate with the *DHTKD1*-encoded 2-oxoadipate dehydrogenase is shown by the kinetic and structural analyses. The preferred binding of the bulkier ligands to 2-oxoadipate dehydrogenase is mediated by the active site serine residue substituting the tyrosine residue in 2-oxoglutarate dehydrogenase. Glutaryl phosphonate shows comparable efficiency in inhibiting both the 2-oxoadipate and 2-oxoglutarate dehydrogenases *in vitro*. Application of adipoyl phosphonate *in vivo* may be used to reveal physiological significance of the *DHTKD1*-encoded 2-oxoadipate dehydrogenase and fight malignant transformations with overexpressed *DHTKD1*.

## Data Availability Statement

The original contributions presented in the study are included in the article/Supplementary Materials, further inquiries can be directed to the corresponding author/s.

## Ethics Statement

The animal study was reviewed and approved by Bioethics Comittee of Lomonosov Moscow State University.

## Author Contributions

AK and NL synthesized the phosphonate analogs. AA performed the kinetic experiments. MB and AA performed structural analysis. VB and AA analyzed the results and wrote the manuscript. All authors reviewed and edited the manuscript.

## Conflict of Interest

The authors declare that the research was conducted in the absence of any commercial or financial relationships that could be construed as a potential conflict of interest.

## Abbreviations

AP: adipoyl phosphonate
DHTKD1: dehydrogenase E1 and transketolase domain-containing protein 1
GP: glutaryl phosphonate
OA: 2-oxoadipate
OADH: 2-oxoadipate dehydrogenase
OG: 2-oxoglutarate
OGDH: 2-oxoglutarate dehydrogenase
OGDHL: 2-oxoglutarate-dehydrogenase-like protein
PDH: pyruvate dehydrogenase
SP: succinyl phosphonate
TESP: triethyl succinyl phosphonate
TEGP: triethyl glutaryl phosphonate
ThDP: thiamine diphosphate
TMAP: trimethyl adipoyl phosphonate.
