# Supplementary material for: Selective Inhibition of 2-Oxoglutarate and 2-Oxoadipate Dehydrogenases by the Phosphonate Analogs of Their 2-Oxo Acid Substrates
Source: Front Chem. 2021 Jan 12;8:596187. doi: 10.3389/fchem.2020.596187 (PMC7835950; doi:10.3389/fchem.2020.596187)
Supplement: Supplementary file 1 [file Image_1.pdf]

A

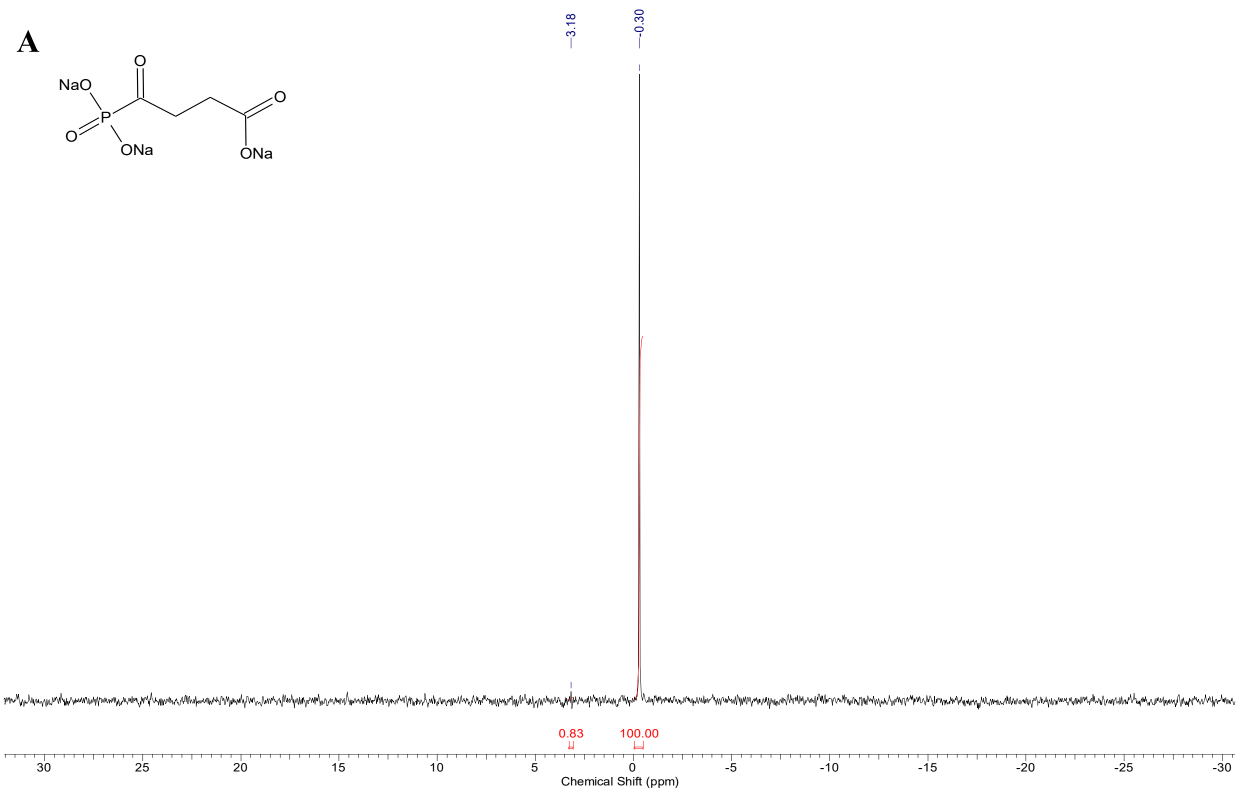

NaO
O
ONa
P
O
ONa
3.18
-0.30
0.83
100.00
Chemical Shift (ppm)

B

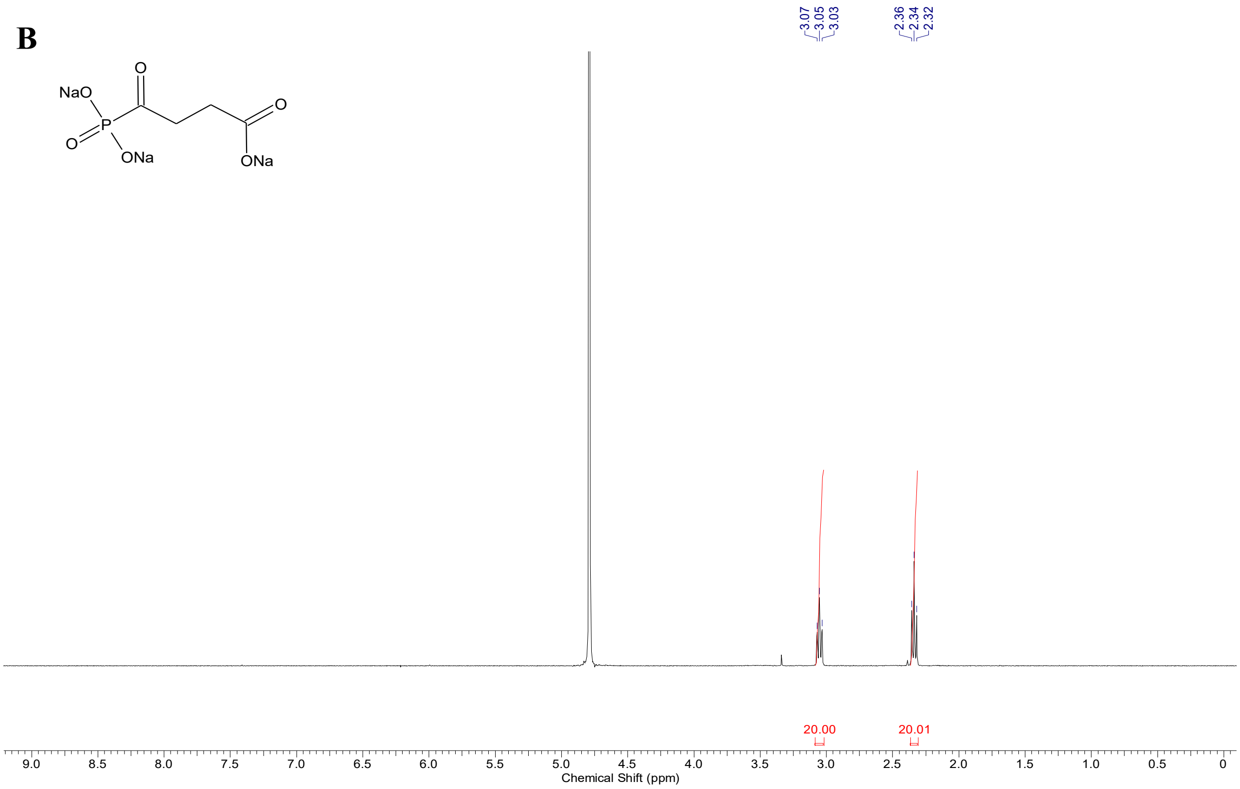

NaO
O
ONa
P
O
ONa
3.07
3.05
3.03
2.36
2.34
2.32
20.00
20.01
Chemical Shift (ppm)

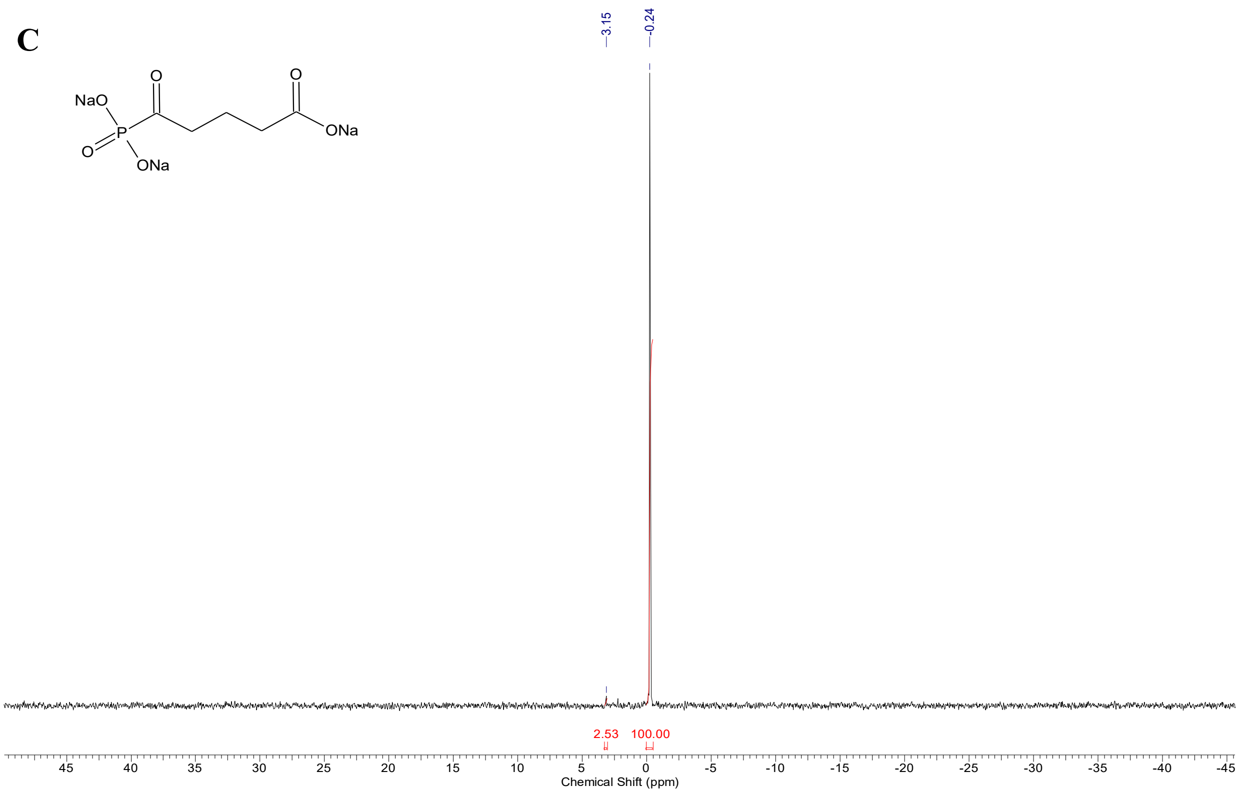

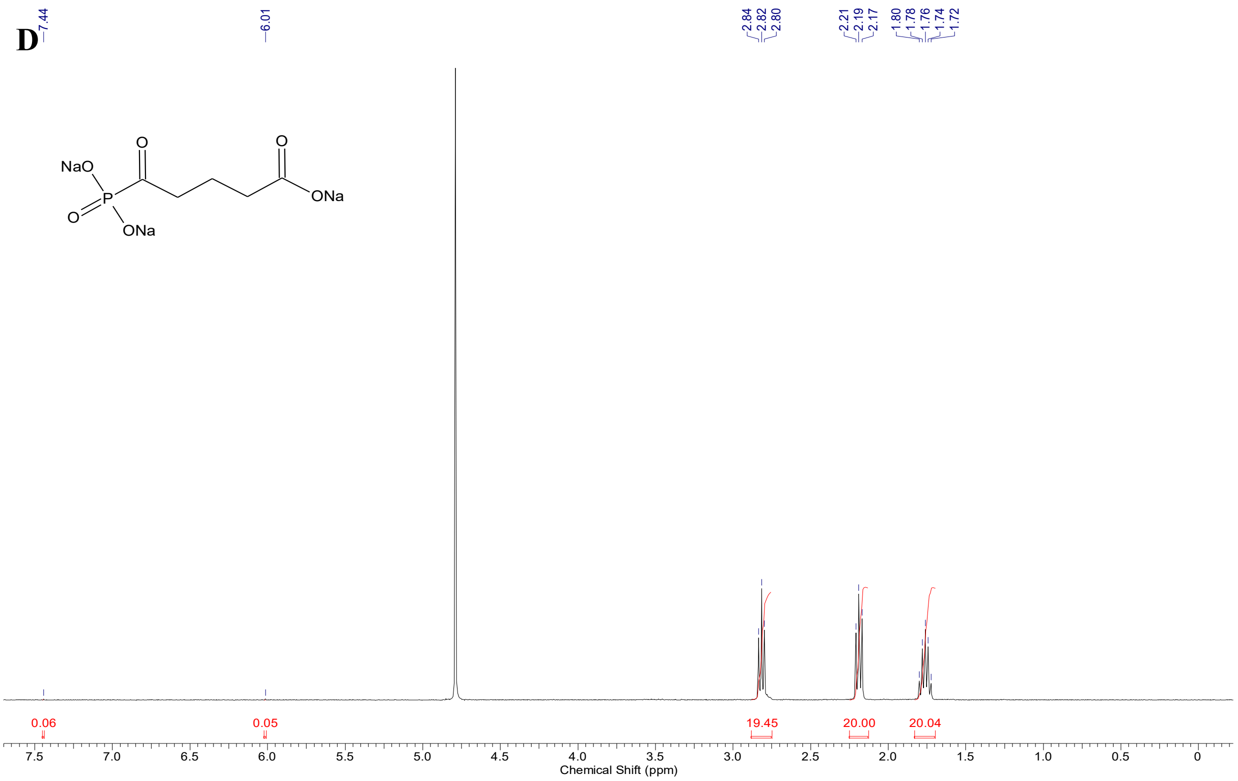

This is a provisional file, not the final typeset article

E

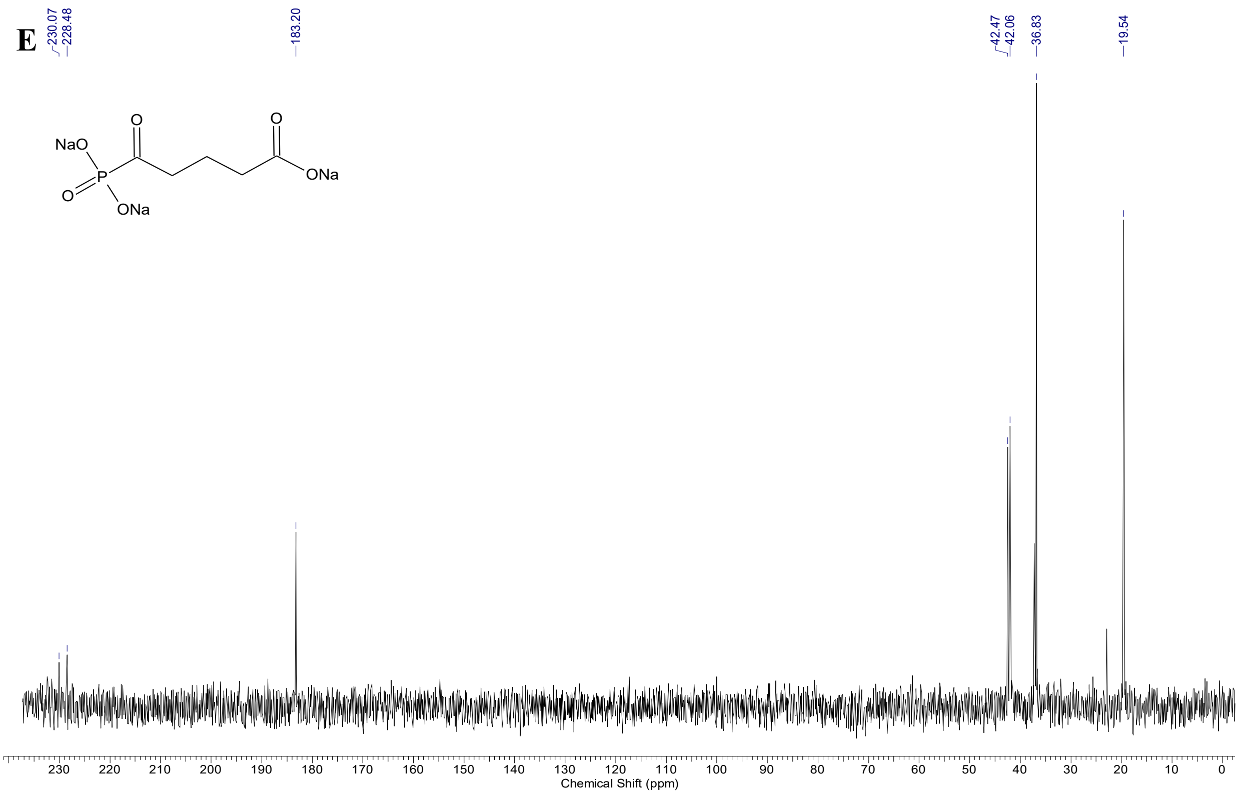
230.07
228.48
183.20
42.47
42.06
36.83
19.54
NaO
O
P
O
ONa
O
ONa
Chemical Shift (ppm)

F

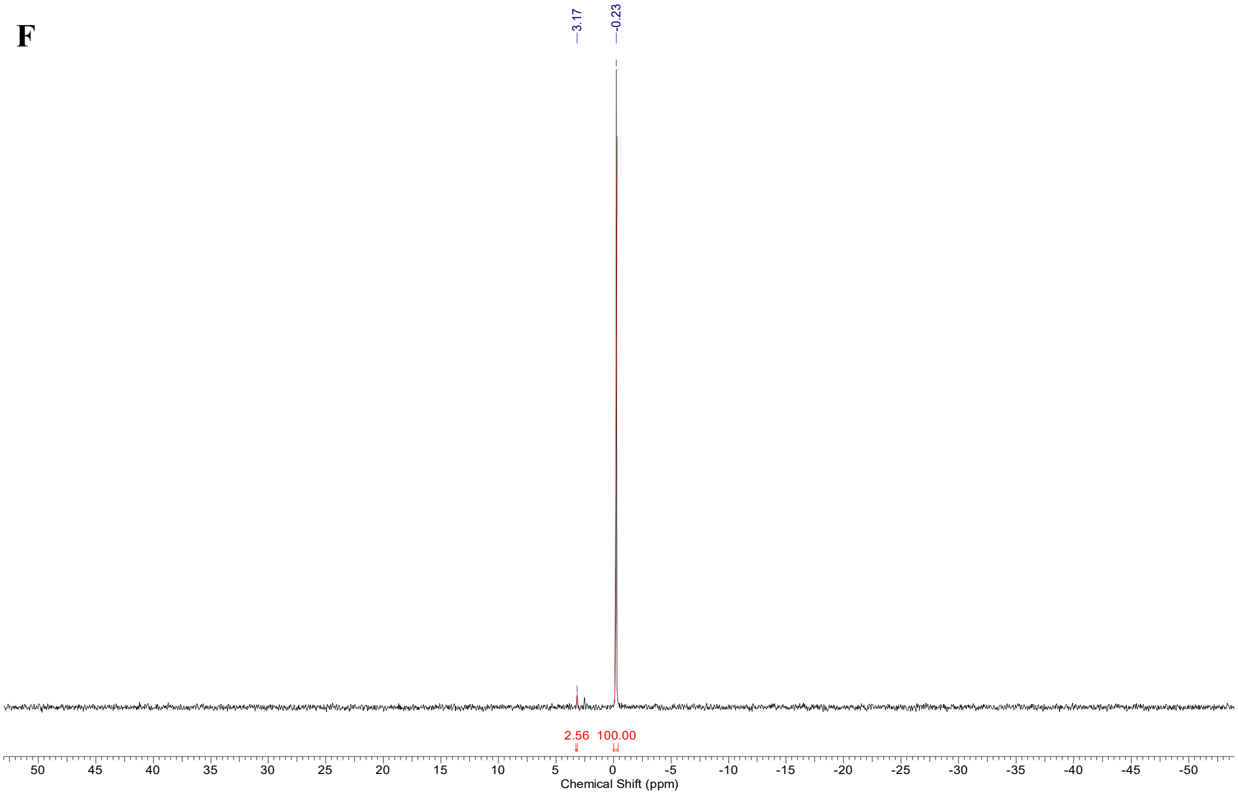
3.17
0.23
2.56
100.00
Chemical Shift (ppm)

G

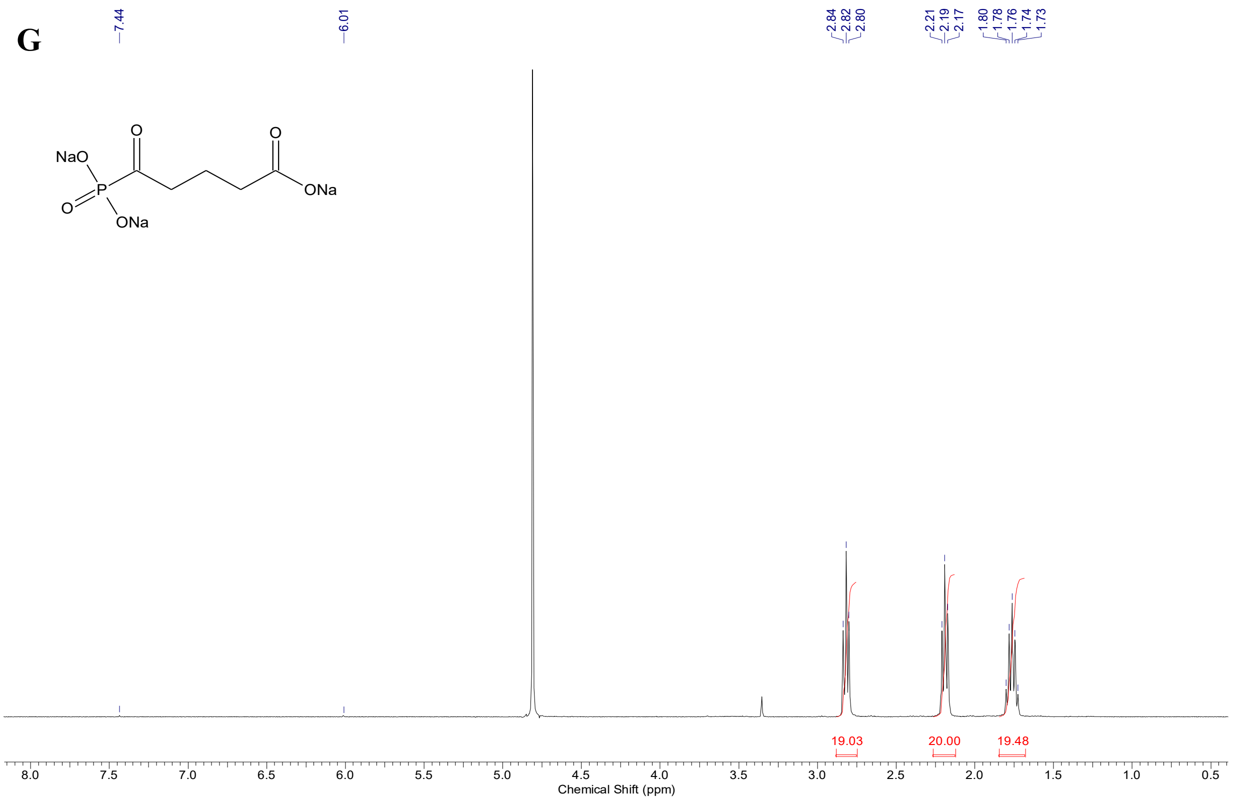
7.44
6.01
2.84
2.82
2.80
2.21
2.19
2.17
1.80
1.78
1.76
1.74
1.73
NaO
O
O
P
O
ONa
ONa
19.03
20.00
19.48
Chemical Shift (ppm)

H

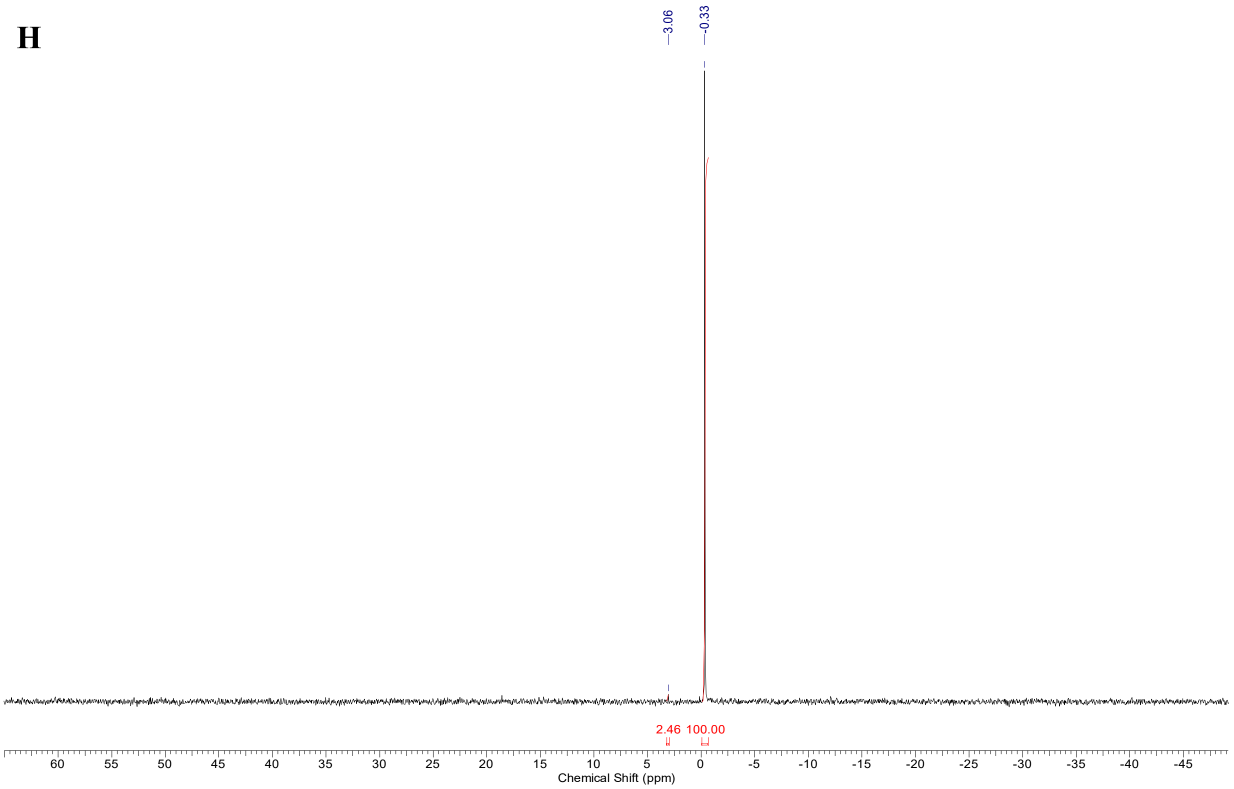
3.06
-0.33
2.46
100.00
Chemical Shift (ppm)

I

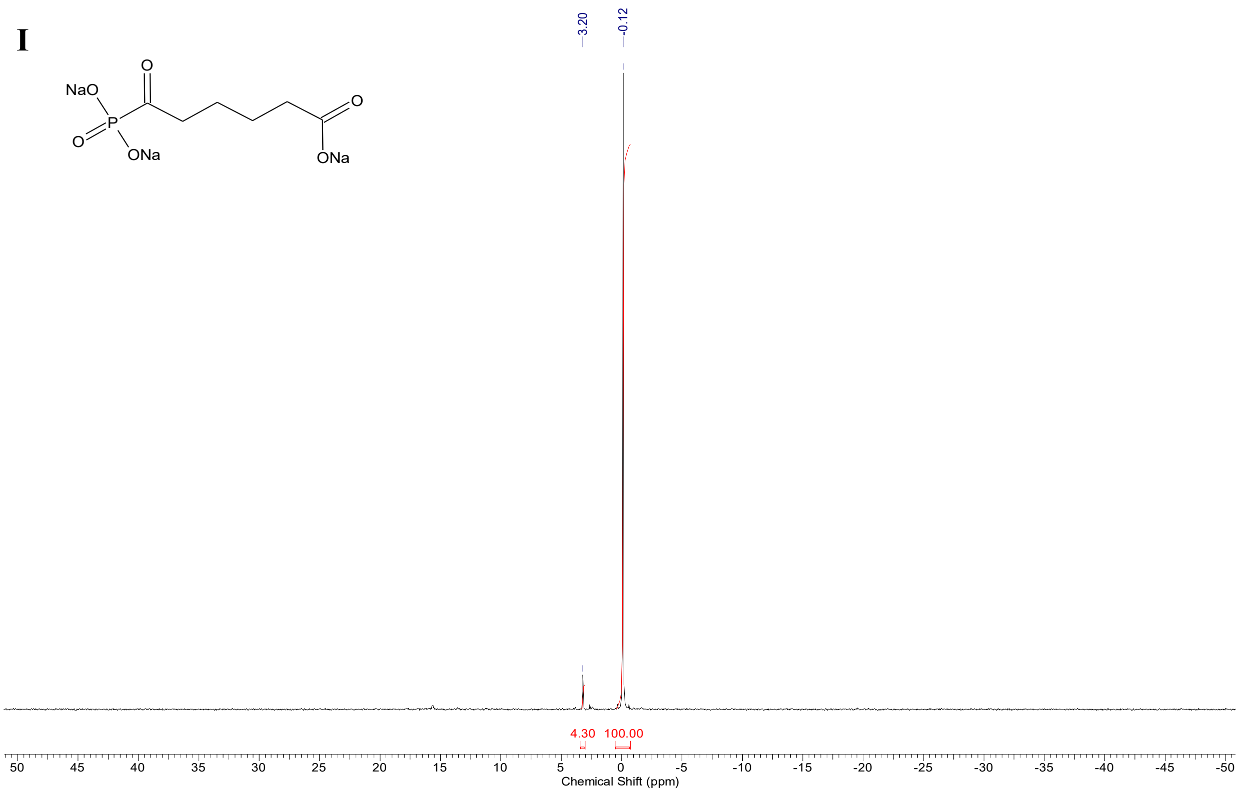
NaO
O
P
O
ONa
O
ONa
3.20
0.12
4.30
100.00
50 45 40 35 30 25 20 15 10 5 0 -5 -10 -15 -20 -25 -30 -35 -40 -45 -50
Chemical Shift (ppm)

J

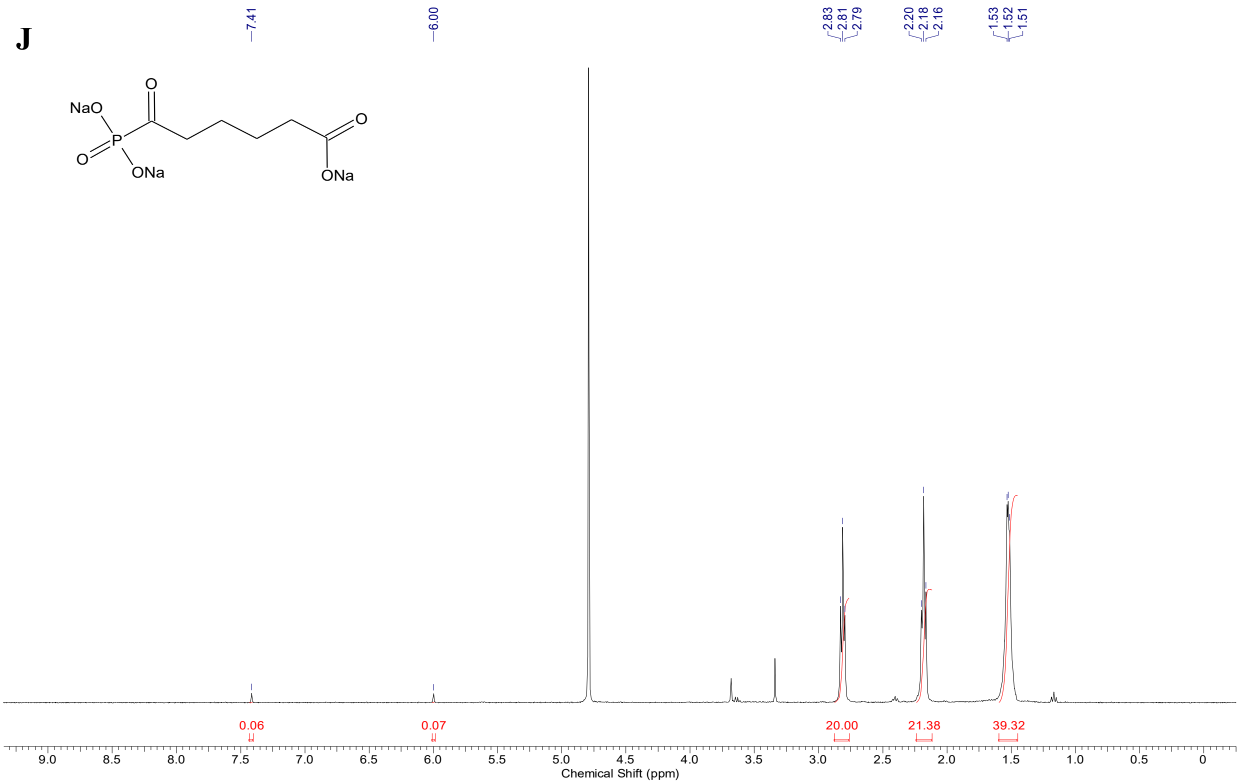
NaO
O
P
O
ONa
O
ONa
7.41
6.00
2.83
2.81
2.79
2.20
2.18
2.16
1.53
1.52
1.51
0.06
0.07
20.00
21.38
39.32
9.0 8.5 8.0 7.5 7.0 6.5 6.0 5.5 5.0 4.5 4.0 3.5 3.0 2.5 2.0 1.5 1.0 0.5 0
Chemical Shift (ppm)

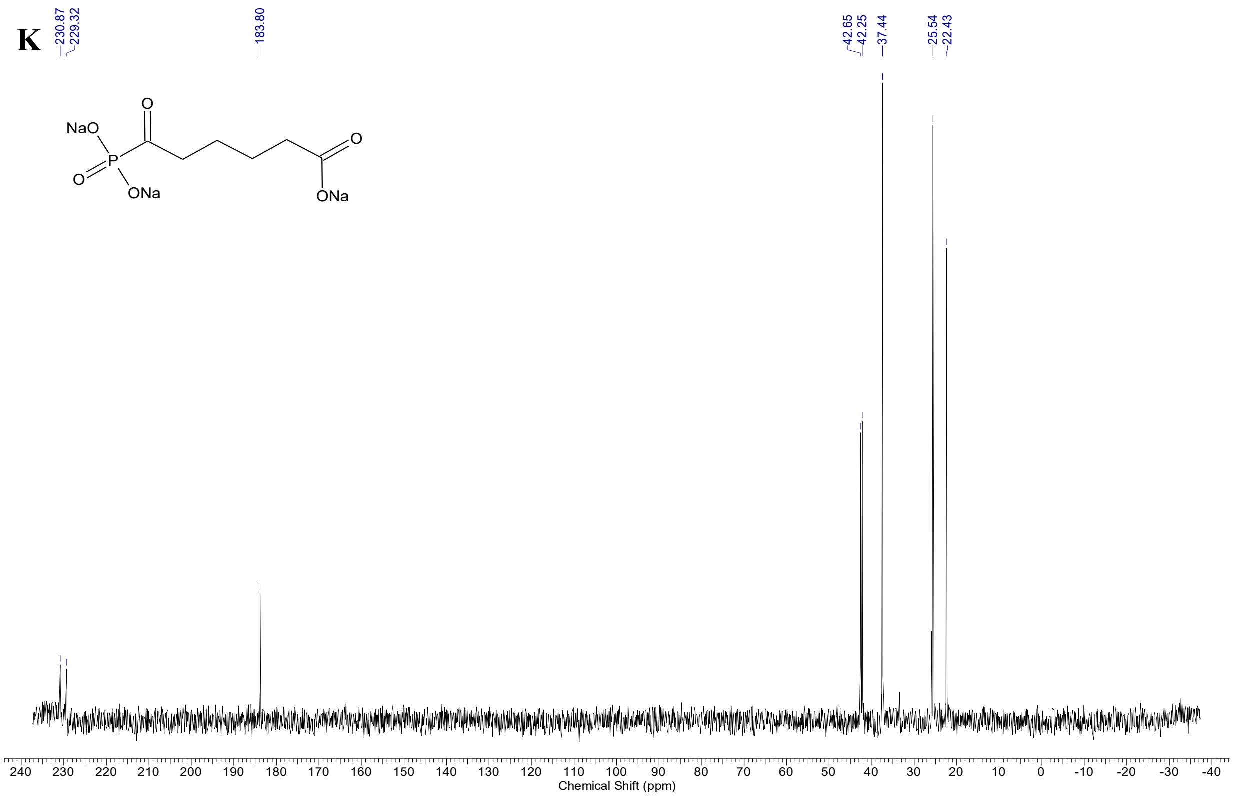

11

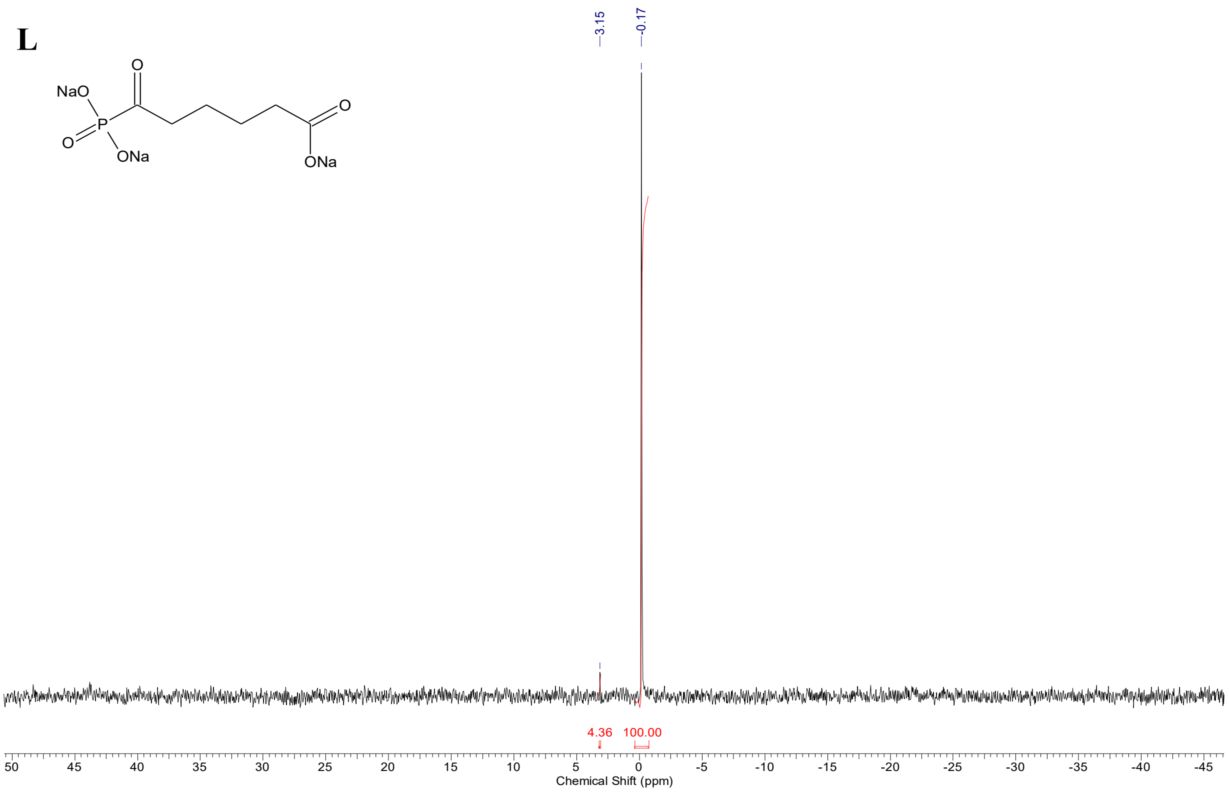

12

This is a provisional file, not the final typeset article

M

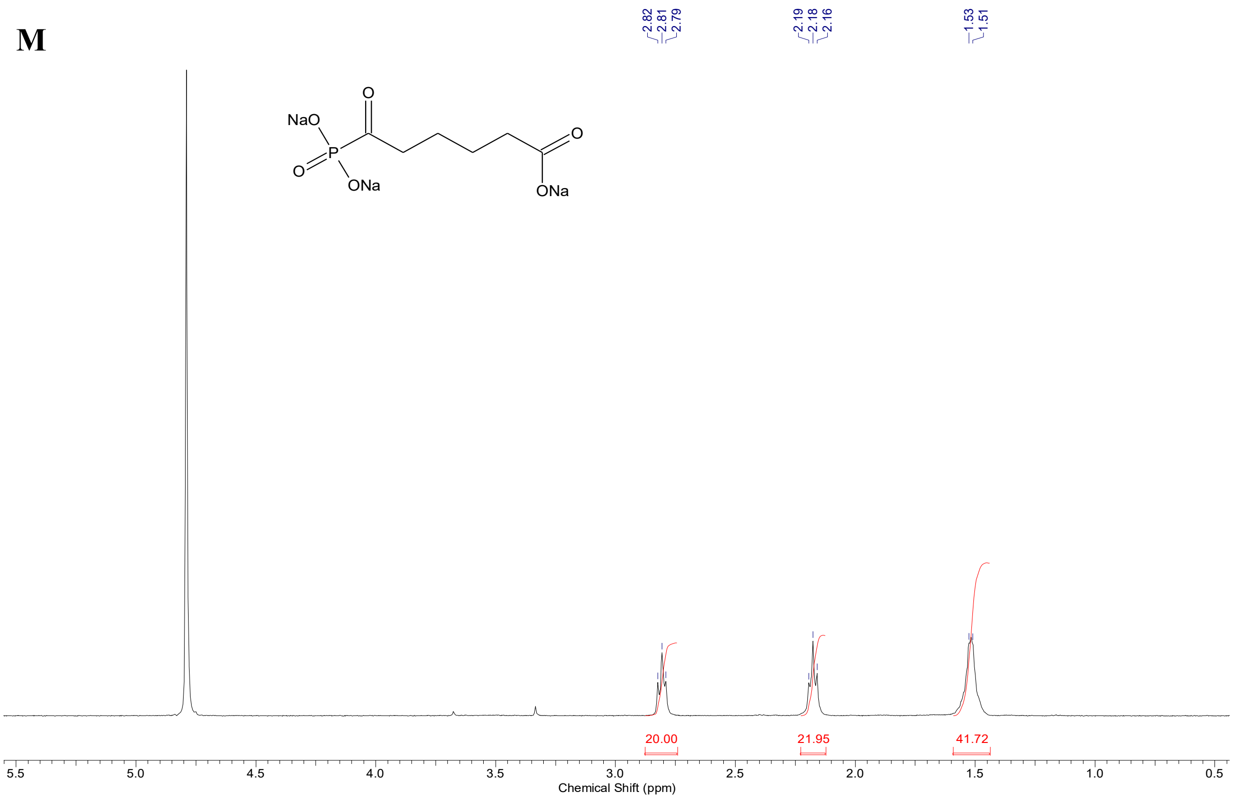

N

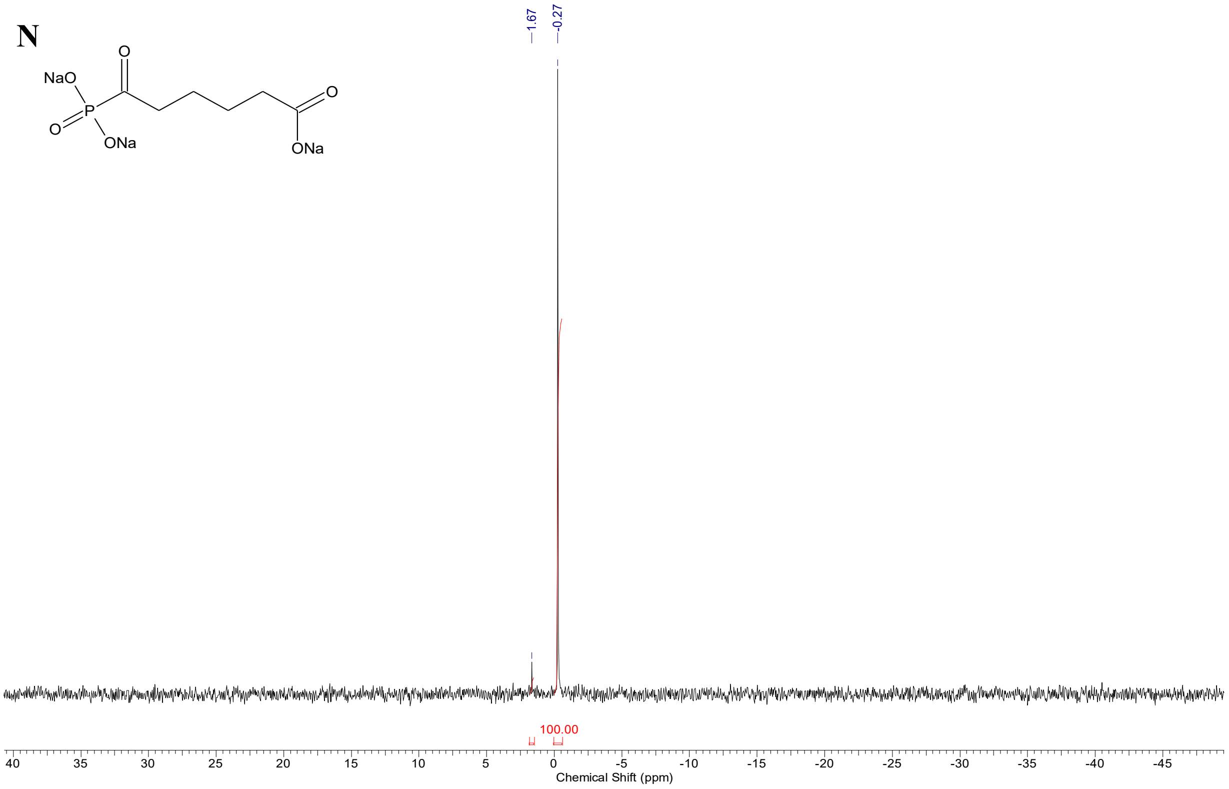

Supplementary Figure 1. NMR analysis of the stability of the 2-oxo phosphonates in the form of trisodium salts. The ^{31}P (A, C, F, H-I, L, N), ^{1}H (B, D, G, J, M) and ^{13}C (E, K) spectra of succinyl (SP; A-B), glutaryl (GP; C-H) and adipoyl (AP; I-N) phosphonates were obtained right after the synthesis (A-E, I-K),

after the shelf storage for 6 months (F-G, L-M) or after the storage of 0.2 M aqueous solutions at -20 °C for 2 months (H, N).

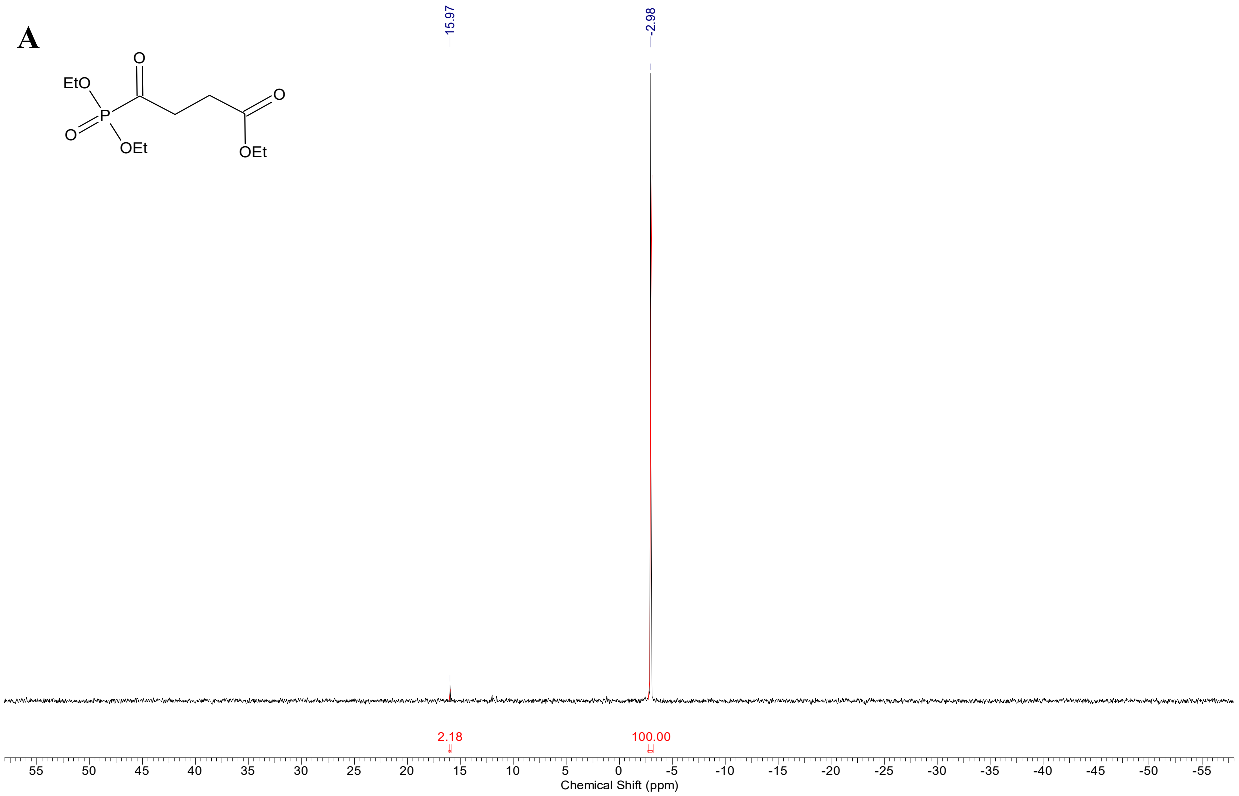

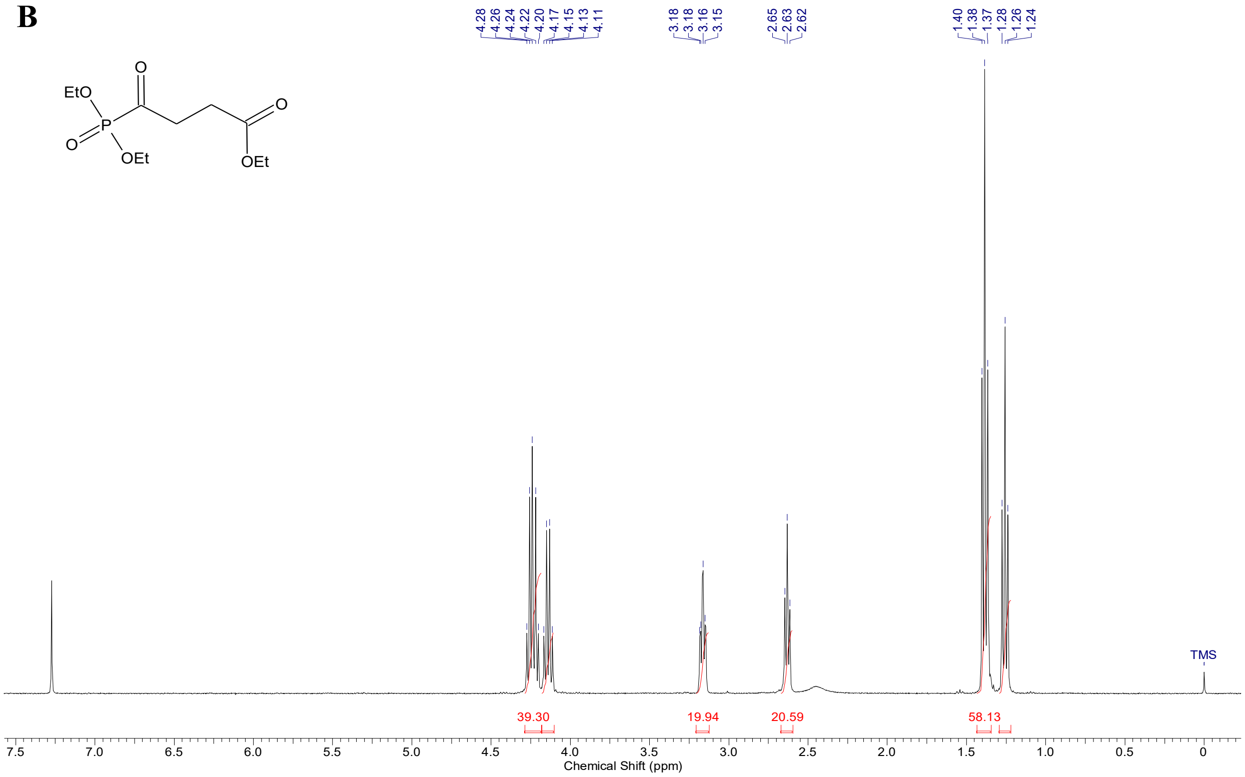

This is a provisional file, not the final typeset article

C

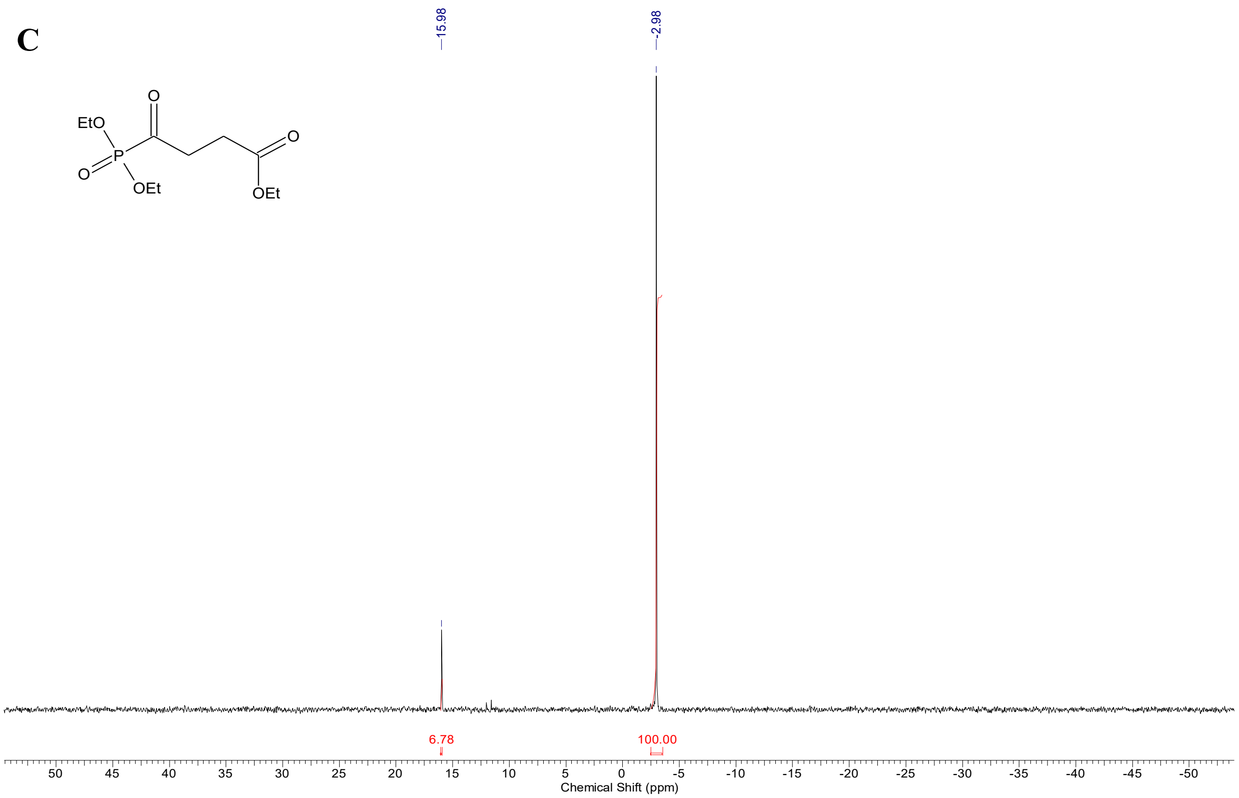
15.98
2.98
EtO
O
P
OEt
OEt
6.78
100.00
Chemical Shift (ppm)

D

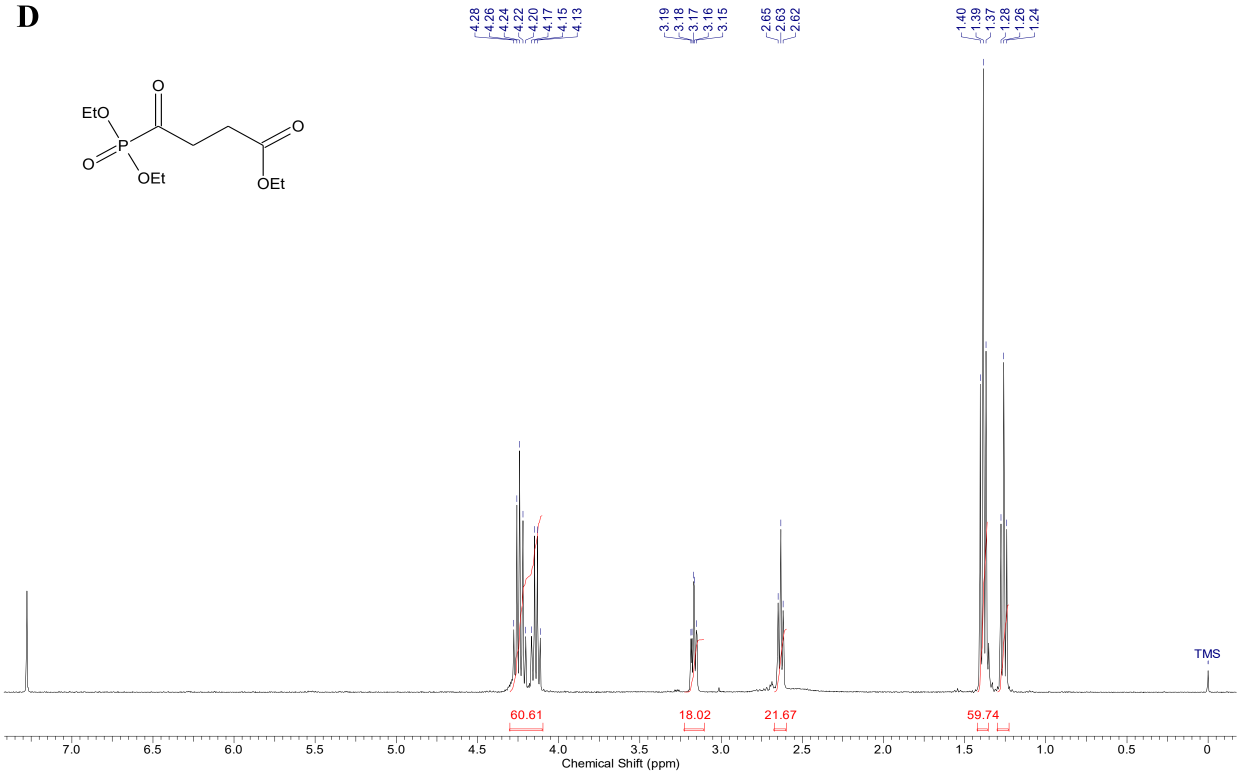
4.28 4.26 4.24 4.22 4.20 4.17 4.15 4.13
3.19 3.18 3.17 3.16 3.15
2.65 2.63 2.62
1.40 1.39 1.37 1.28 1.26 1.24
EtO
O
P
OEt
OEt
TMS
60.61
18.02
21.67
59.74
Chemical Shift (ppm)

E

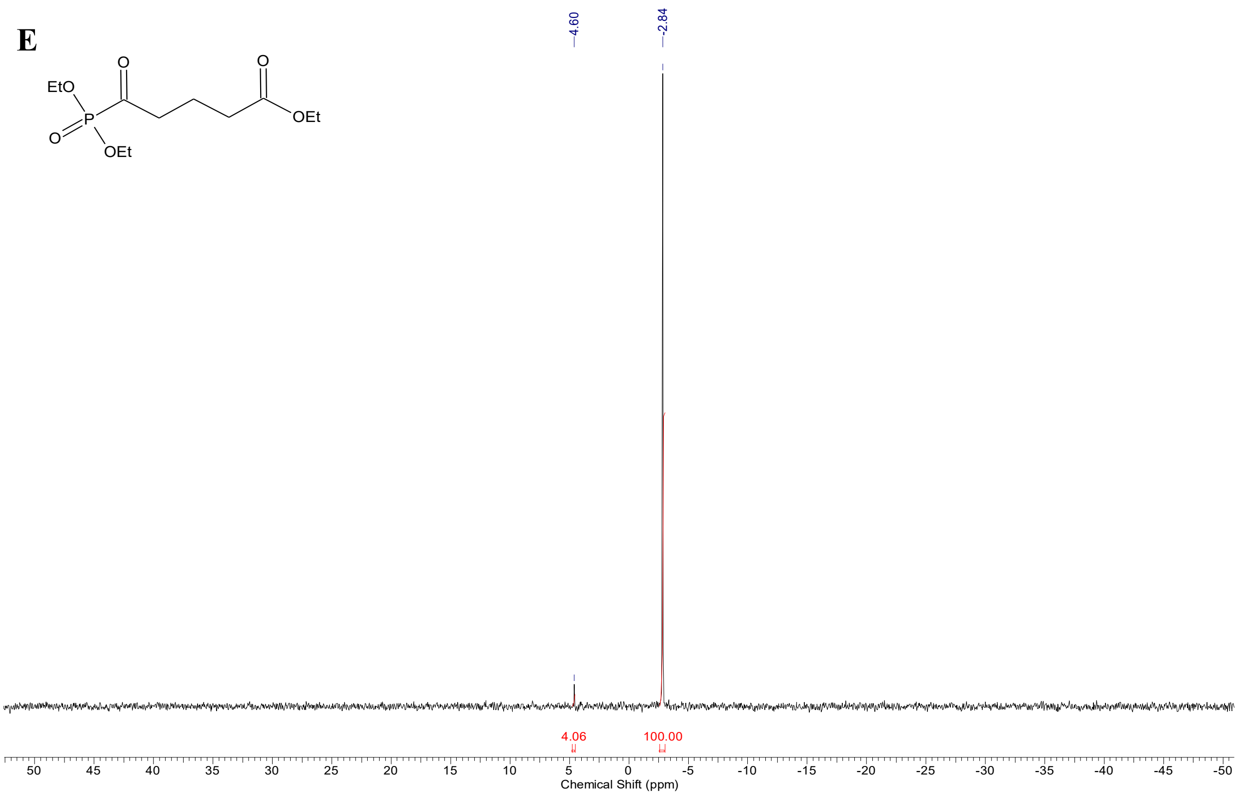

F

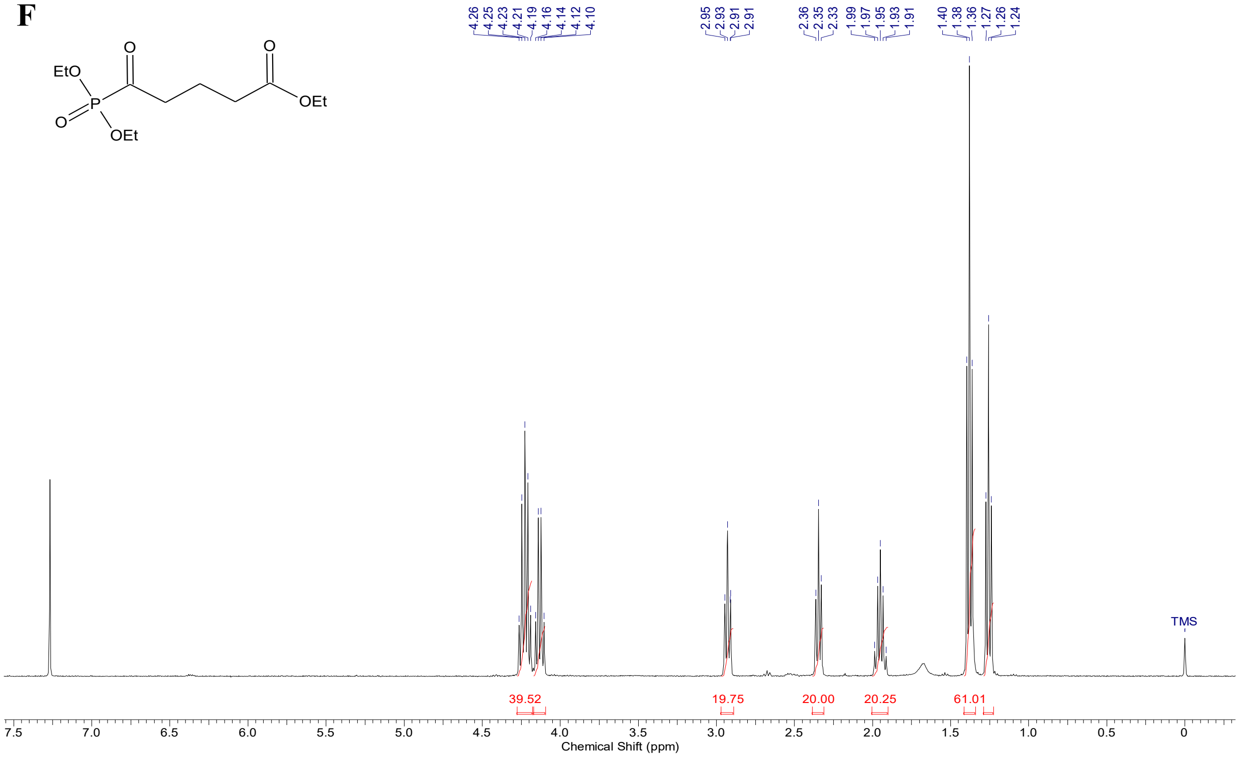

This is a provisional file, not the final typeset article

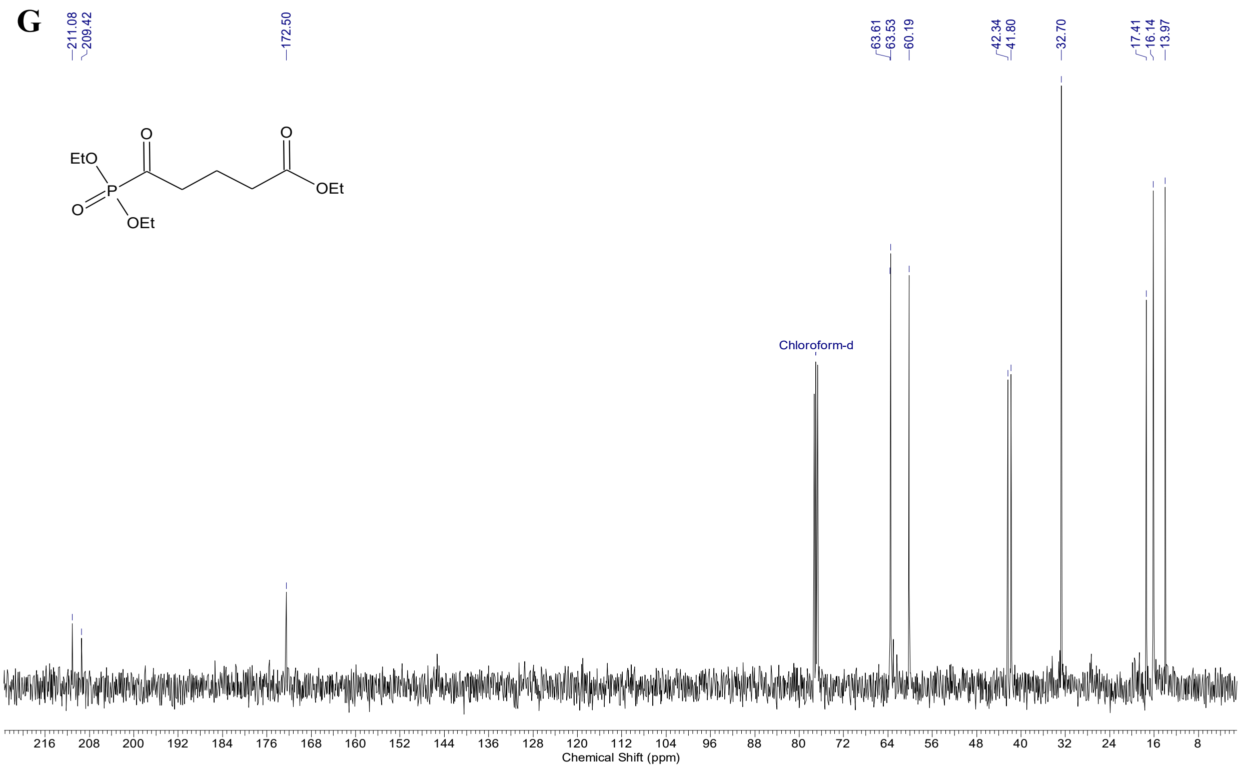
G
211.08
209.42
172.50
63.61
63.53
60.19
42.34
41.80
32.70
17.41
16.14
13.97
EtO
O
P
OEt
O
O
OEt
Chloroform-d
216 208 200 192 184 176 168 160 152 144 136 128 120 112 104 96 88 80 72 64 56 48 40 32 24 16 8
Chemical Shift (ppm)

26

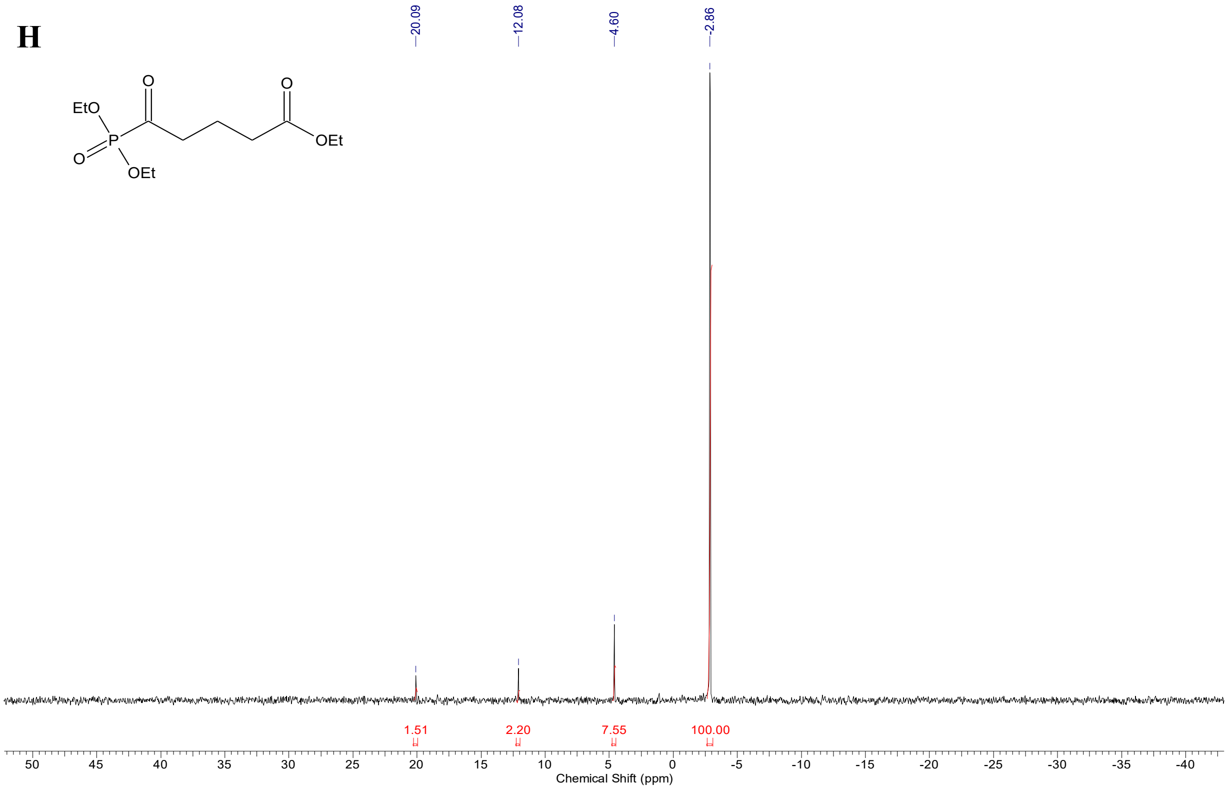
H
20.09
12.08
4.60
-2.86
EtO
O
P
OEt
O
O
OEt
1.51
2.20
7.55
100.00
50 45 40 35 30 25 20 15 10 5 0 -5 -10 -15 -20 -25 -30 -35 -40
Chemical Shift (ppm)

27

I

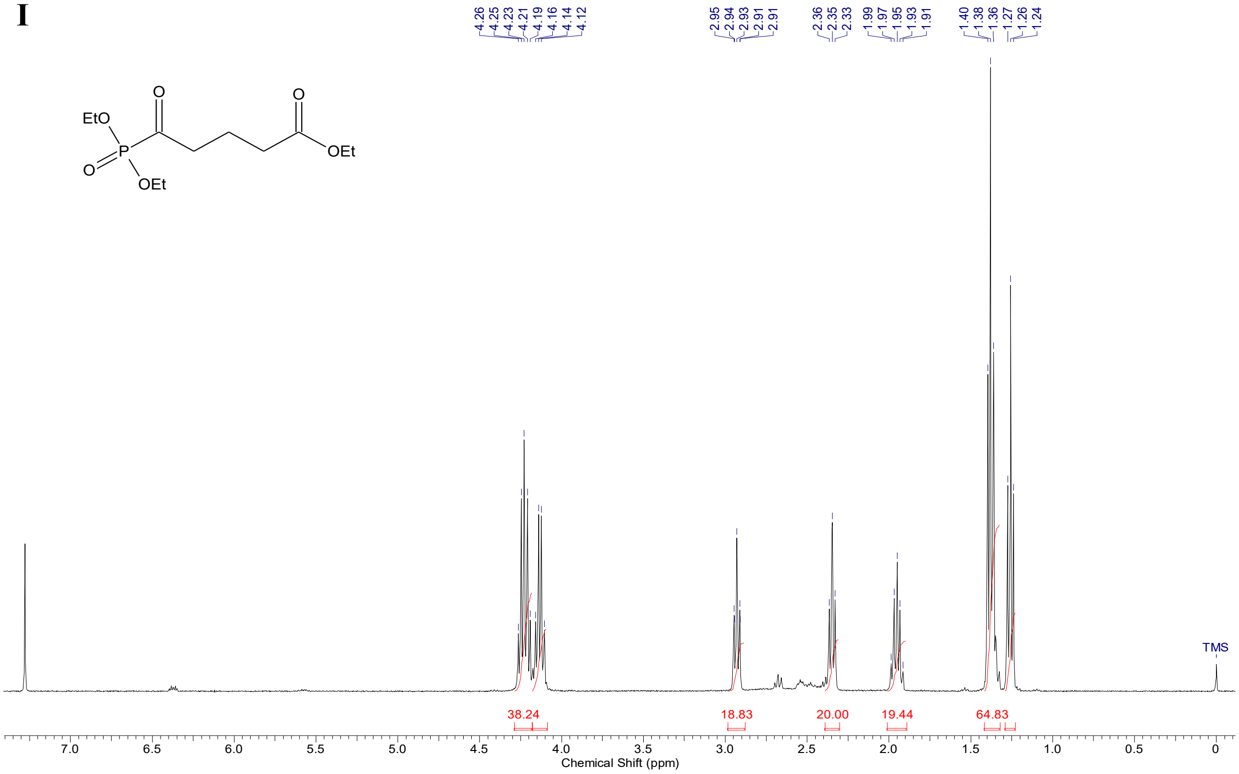

J

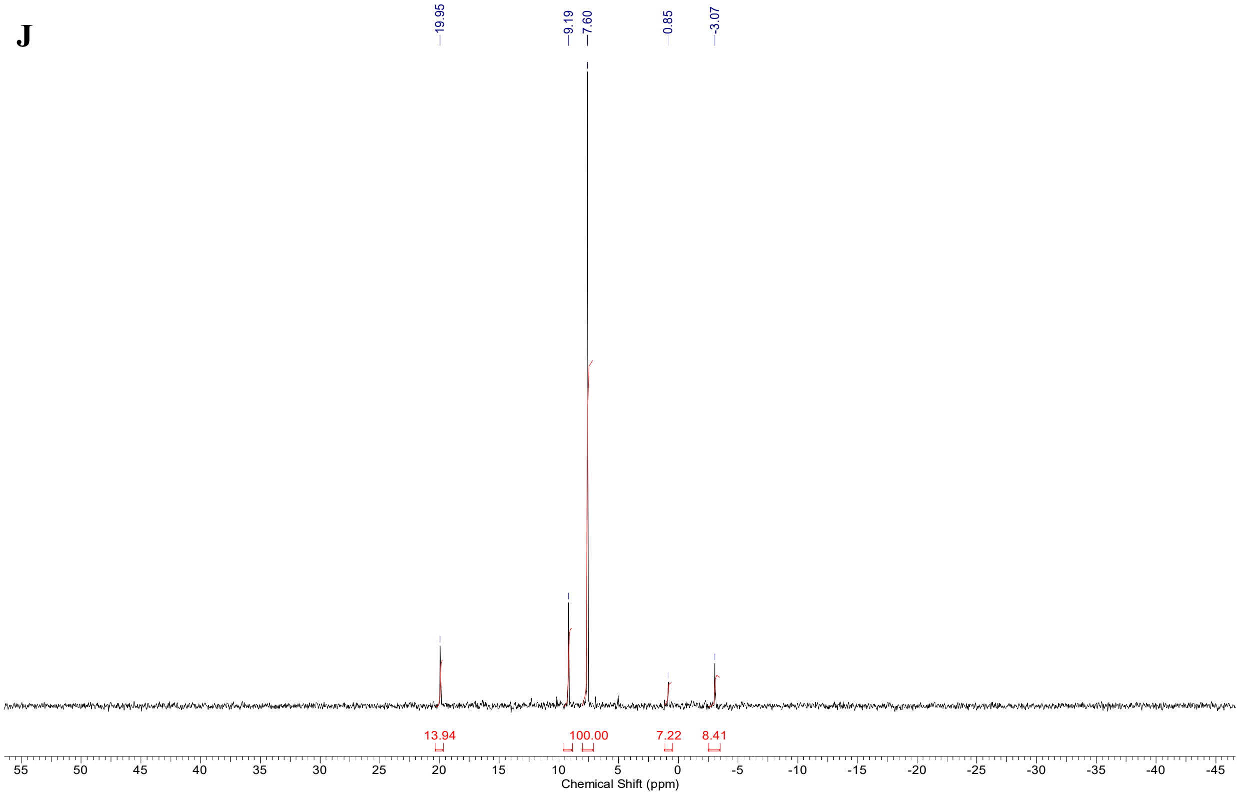

This is a provisional file, not the final typeset article

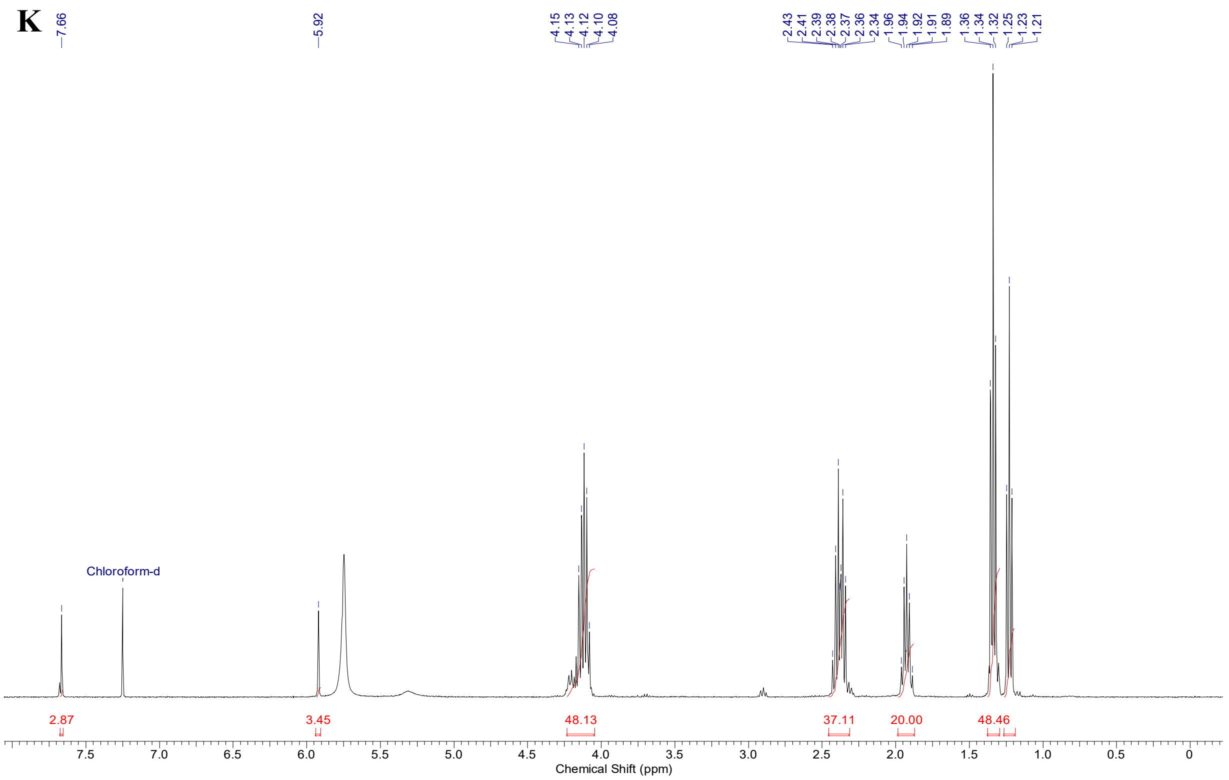
K
7.66
5.92
4.15 4.13 4.12 4.10 4.08
2.43 2.41 2.39 2.38 2.37 2.36 2.34 1.96 1.94 1.92 1.91 1.89 1.36 1.34 1.32 1.25 1.23 1.21
Chloroform-d
2.87
3.45
48.13
37.11
20.00
48.46
Chemical Shift (ppm)

30

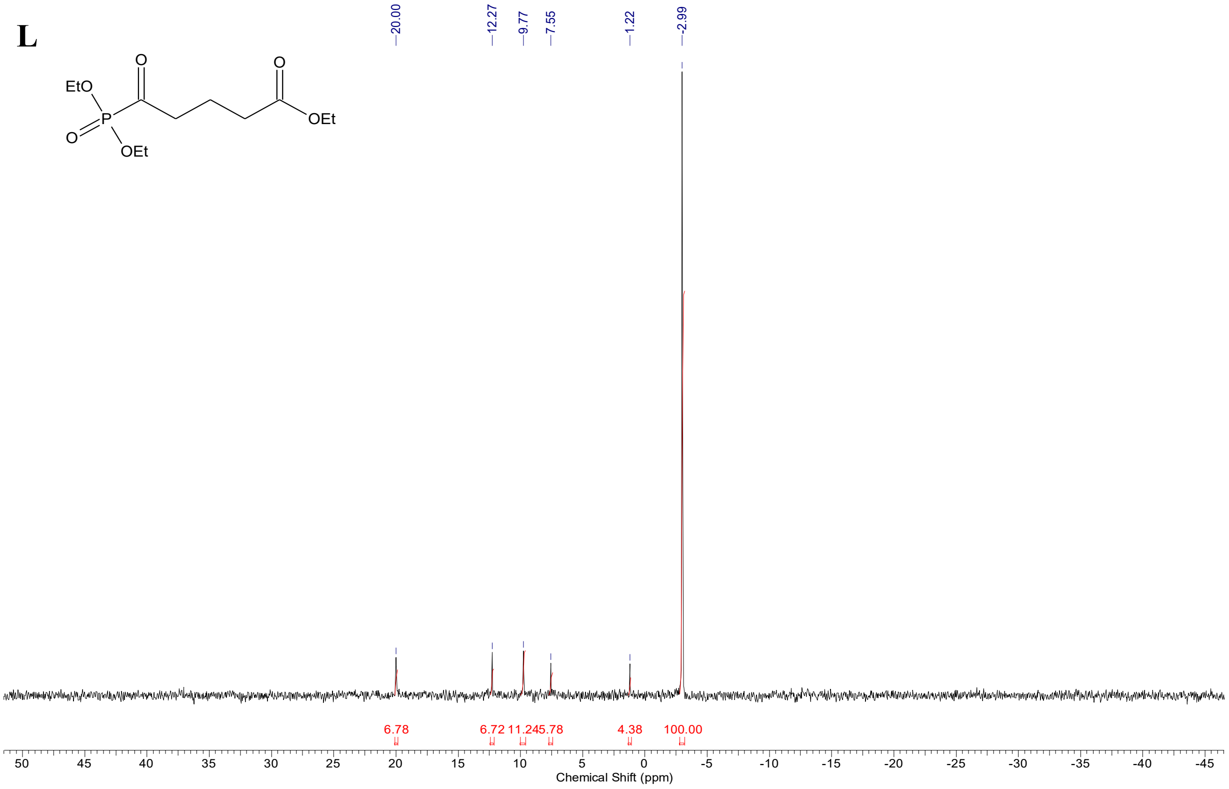
L
20.00
12.27
9.77
7.55
1.22
-2.99
EtO
O
P
OEt
OEt
6.78
6.72 11.24 5.78
4.38
100.00
Chemical Shift (ppm)

31

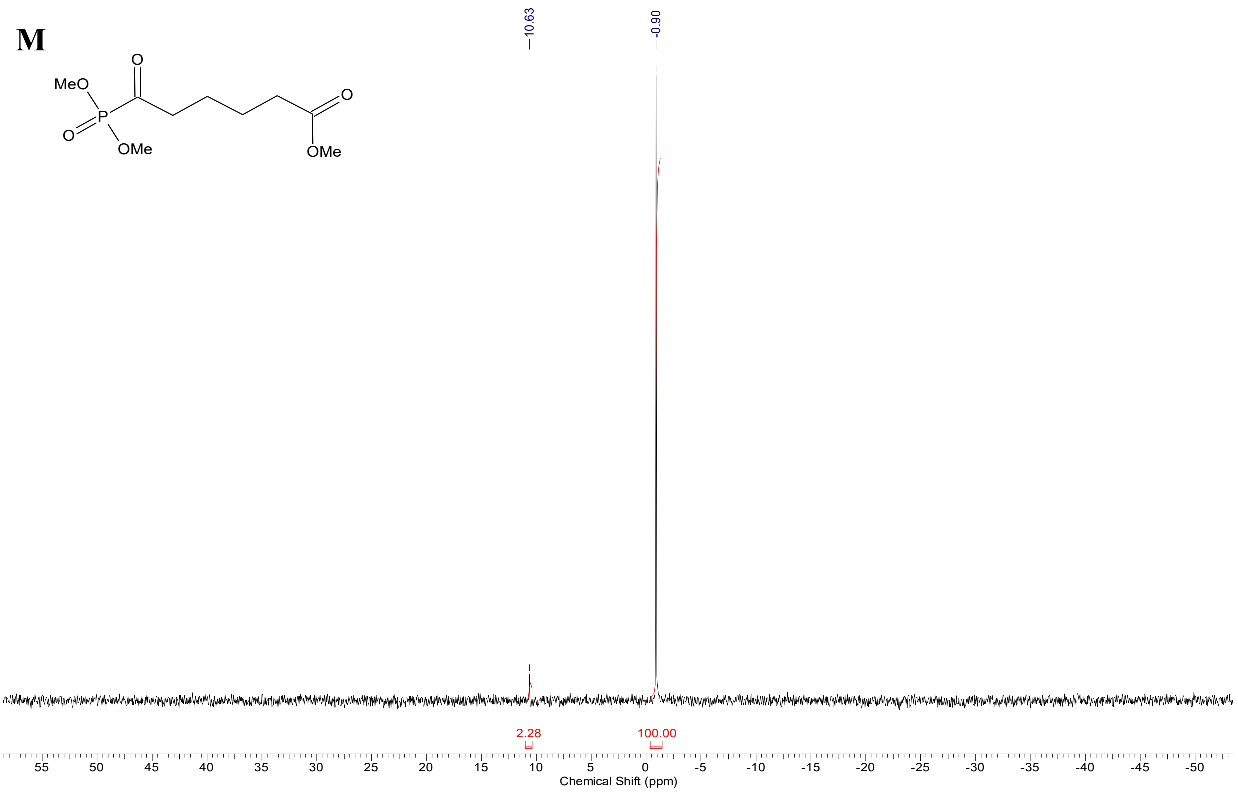

32

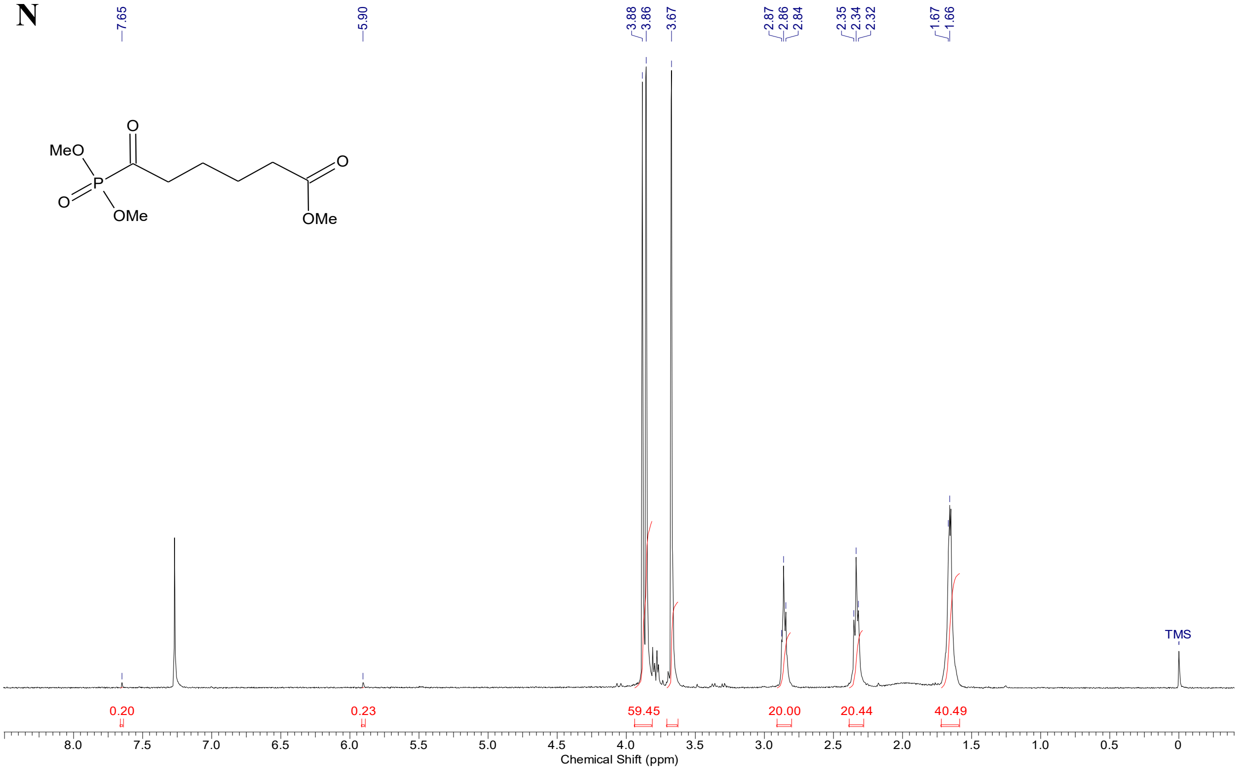

33

This is a provisional file, not the final typeset article

O

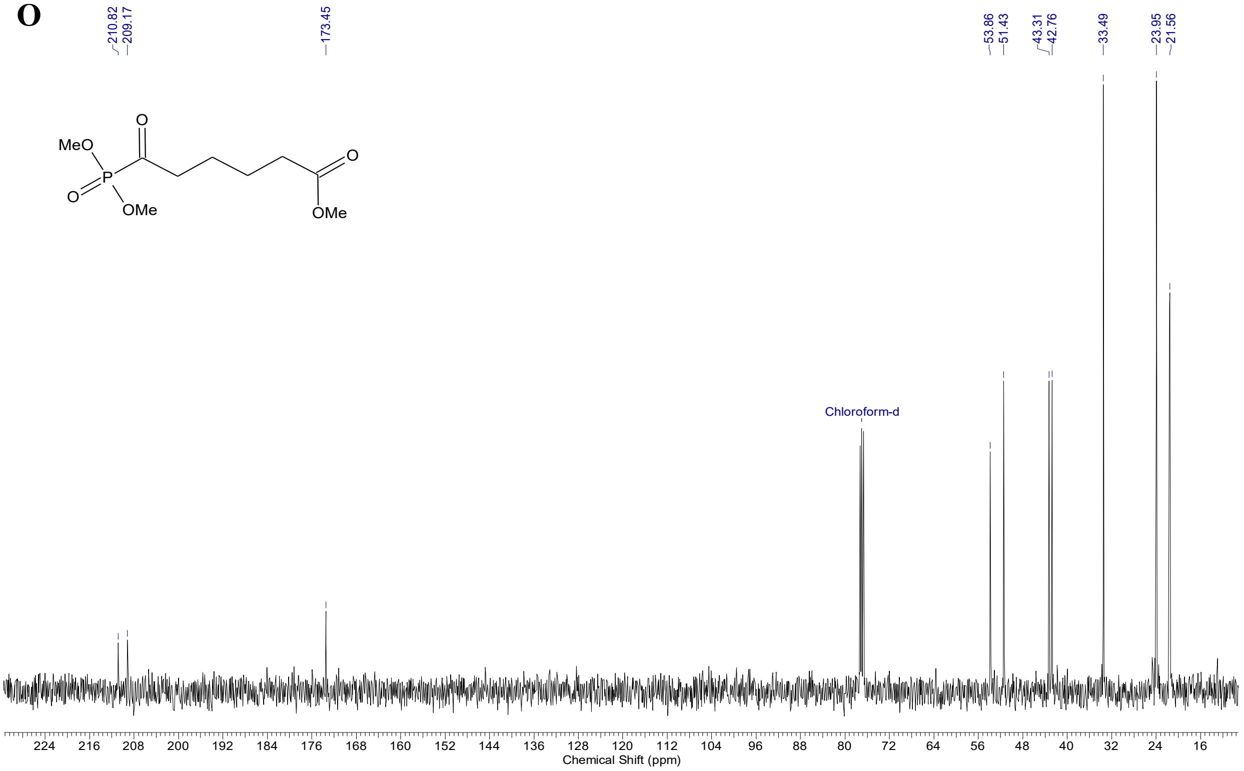
210.82
209.17
173.45
53.86
51.43
43.31
42.76
33.49
23.95
21.56
MeO
O
OMe
OMe
Chloroform-d
Chemical Shift (ppm)

P

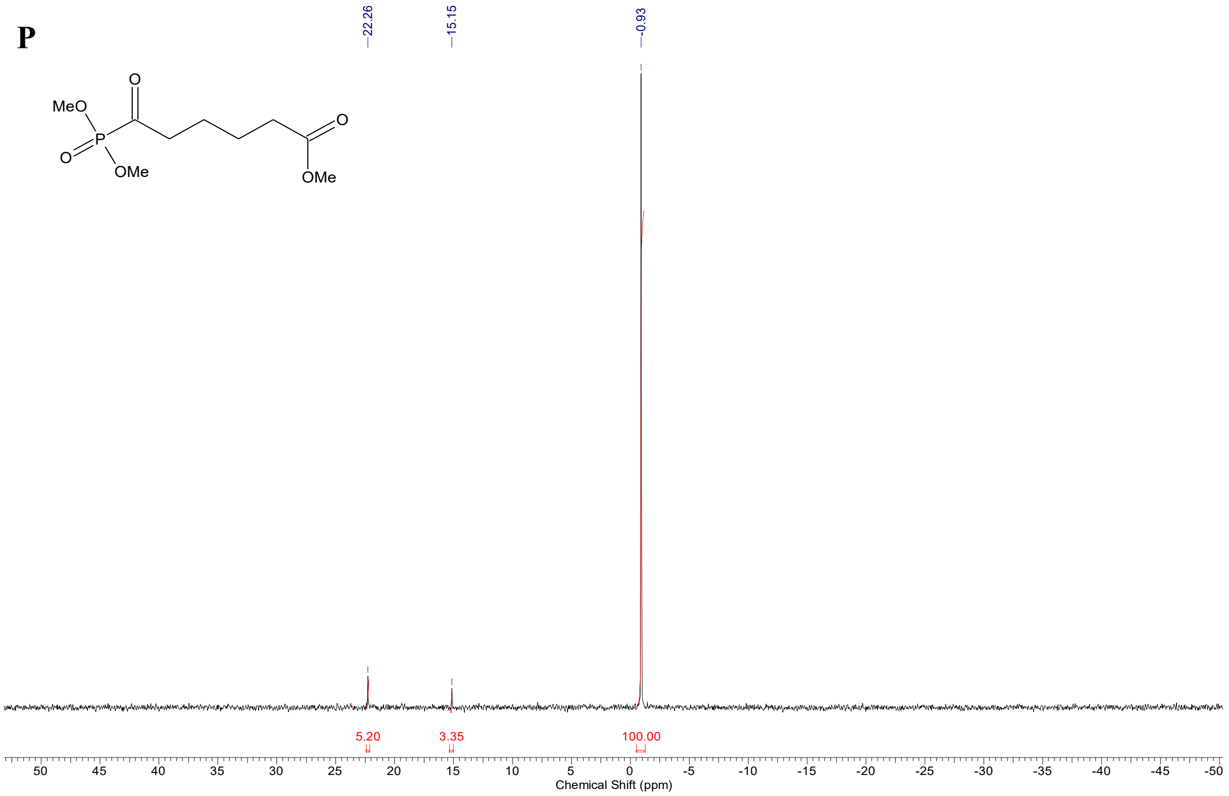
22.26
15.15
-0.93
MeO
O
OMe
OMe
5.20
3.35
100.00
Chemical Shift (ppm)

Q

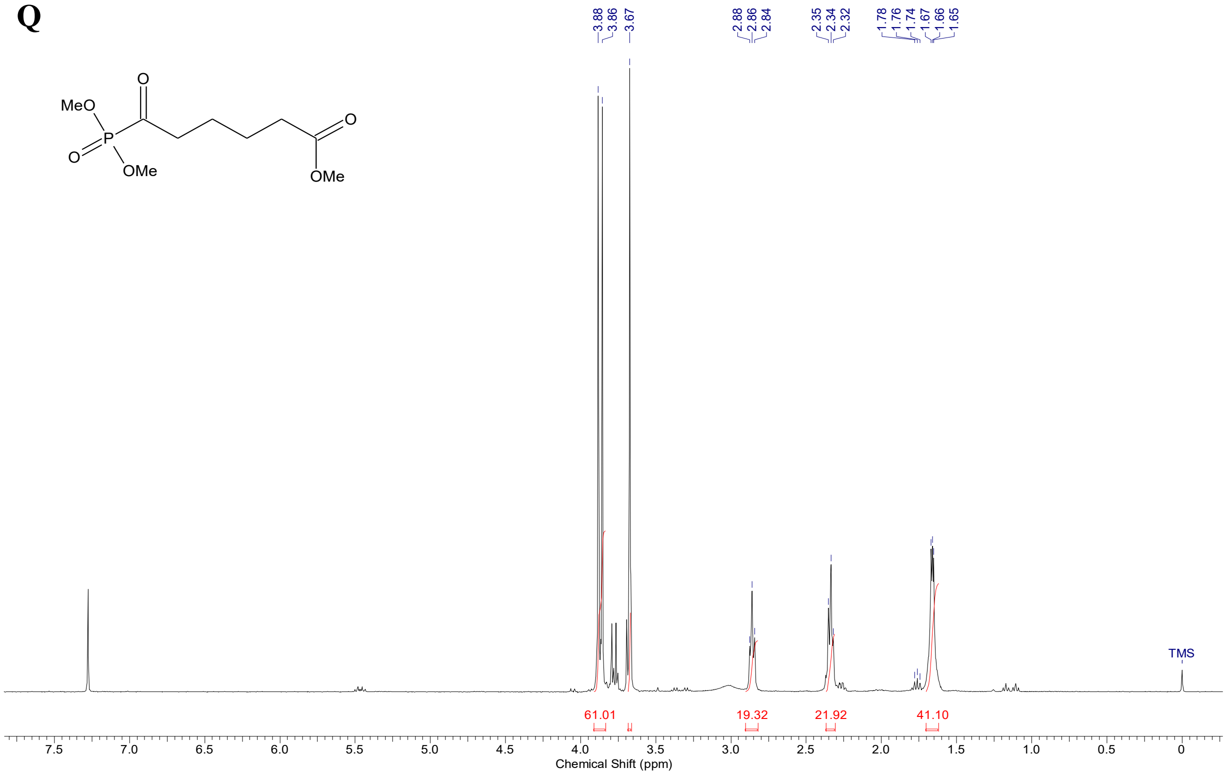

R

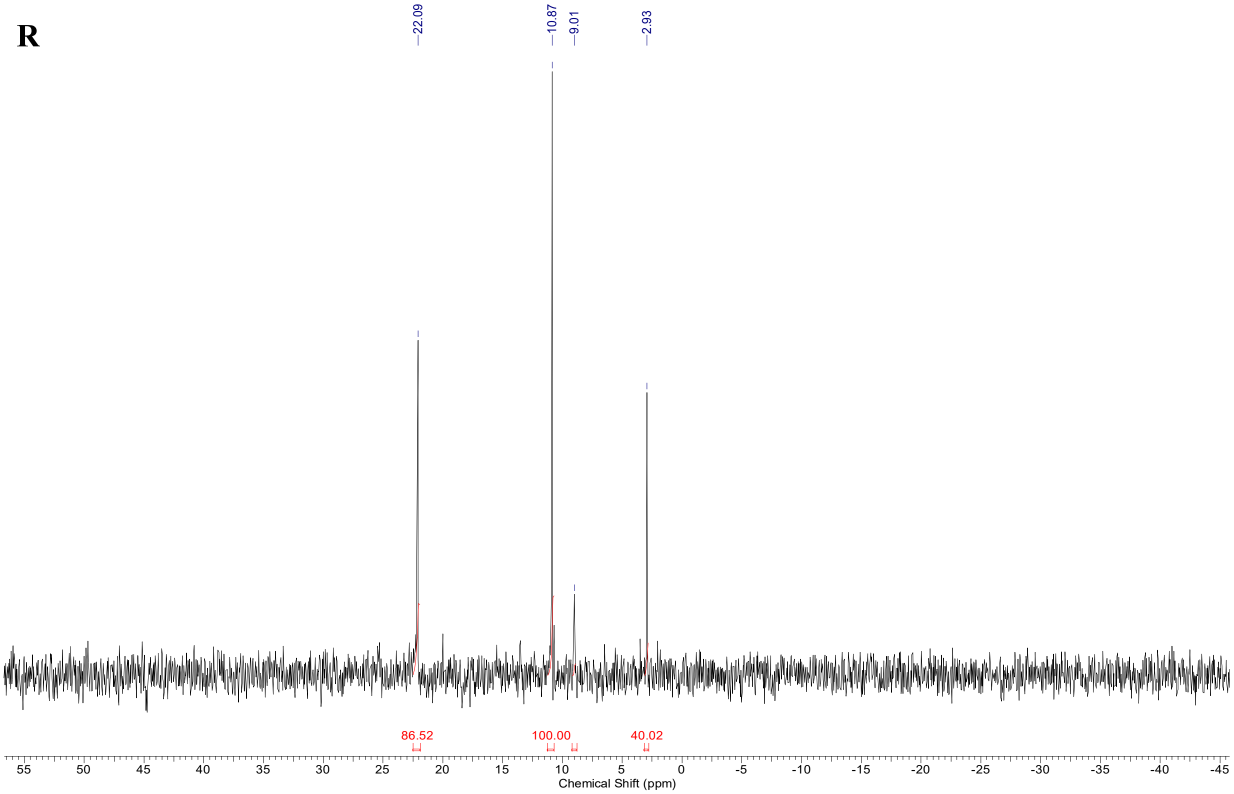

This is a provisional file, not the final typeset article

S

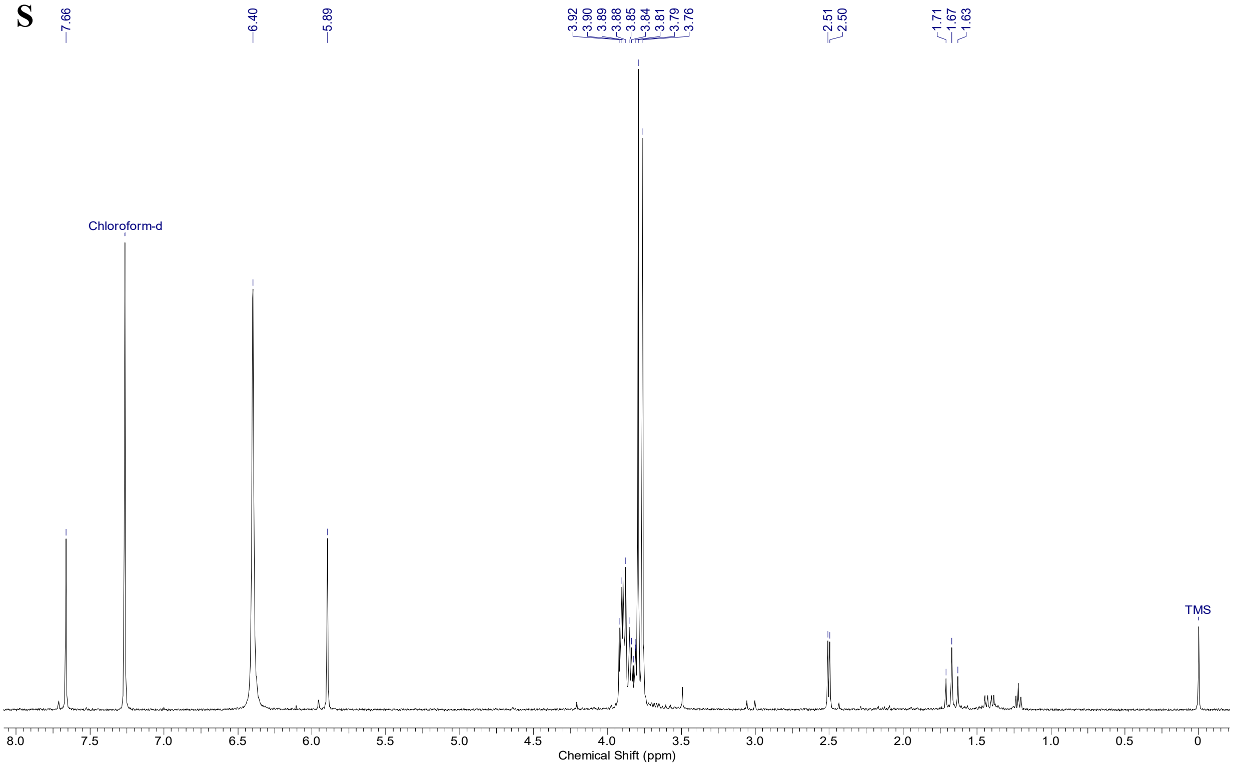

T

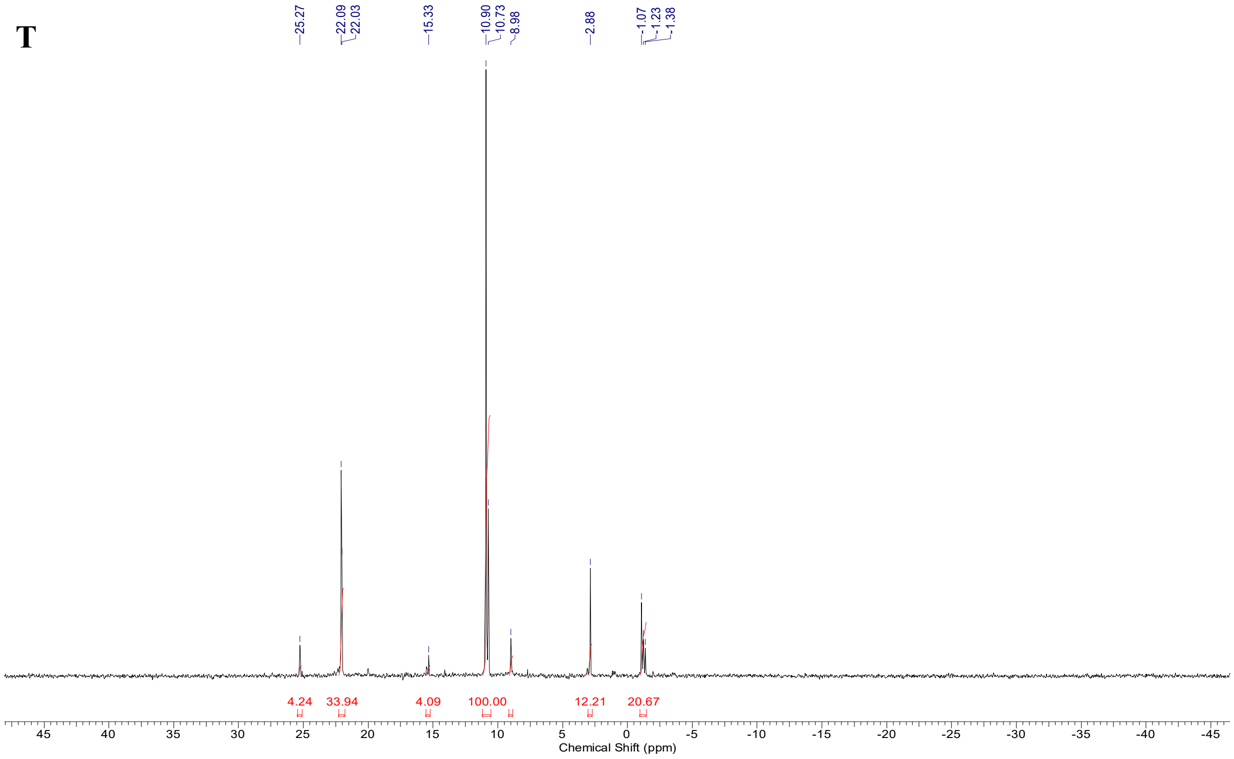

Supplementary Figure 2. NMR analysis of the stability of the 2-oxo phosphonates in their esterified forms. The ^{31}P (A, C, E, H, J, L-M, P, R, T), ^{1}H (B, D, F, I, K, N, Q, S) and ^{13}C (G, O) spectra of triethyl ester of succinyl phosphonate (TESP; A-D), triethyl ester of glutaryl phosphonate (TEGP; E-L) and trimethyl ester of adipoyl phosphonate (TMAP; M-T) were obtained right after the synthesis (A-B, E-G, M-O), after storage

for 6 months under argon (C-D, H-I, P-Q), for 5 days after exposure to air (J-K, R-S) or for 5 days as 0.2 M solutions in water (L, T).

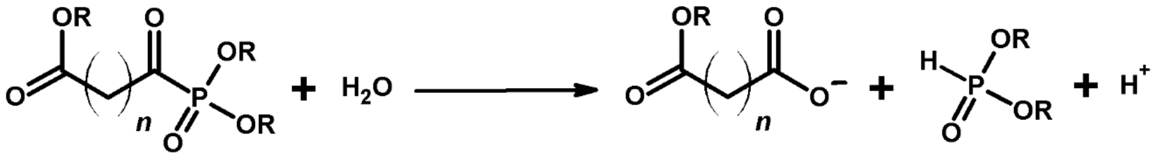

Supplementary Figure 3. Scheme of the decomposition of phosphonate esters during storage. For triethyl succinyl phosphonate: R– = CH_3CH_2–, n = 2; for triethyl glutaryl phosphonate: R– = CH_3CH_2–, n = 3; for trimethyl adipoyl phosphonate: R– = CH_3–, n = 4.

This is a provisional file, not the final typeset article
