# Supplementary material for: Selective Inhibition of 2-Oxoglutarate and 2-Oxoadipate Dehydrogenases by the Phosphonate Analogs of Their 2-Oxo Acid Substrates
Source: Front Chem. 2021 Jan 12;8:596187. doi: 10.3389/fchem.2020.596187 (PMC7835950; doi:10.3389/fchem.2020.596187)
Supplement: Supplementary file 2 [file Table_1.docx]

**Supplementary Table 1. Analysis of the mechanism of the phosphonate inhibition of the OGDH and OADH reactions, catalyzed by the enzyme-enriched preparations from the rat heart and liver.** The experimental dependencies of the enzymatic activities on the 2-oxoglutarate or 2-oxoadipate concentrations at selected concentrations (0, 2, 10, 50, 200 and 1000 µM) of the inhibitors (I): the succinyl (SP), glutaryl (GP) and adipoyl (AP) phosphonates – are approximated by Michaelis-Menten equations to determine the kinetic parameters of the dependencies (*K_m_,* mM and *A_max_,* μmol/min per mg of protein). OADH reaction catalyzed by the enzyme-enriched preparation from liver is analyzed at high (0.1-10 mM) and low (0-0.05 mM) 2-oxoadipate concentration separately to give the parameters for the low-affinity and high-affinity active sites. Data are presented as Mean ± Standard Error of Mean.

| **I** | **[I],**  **µM** | **OGDH reaction** | | | | **OADH reaction** | | | | | |
| --- | --- | --- | --- | --- | --- | --- | --- | --- | --- | --- | --- |
|  |  | **Heart** | | **Liver** | | **Heart** | | **Liver** | | | |
|  |  |  |  |  |  |  |  | **Low-affinity sites** | | **High-affinity sites** | |
|  |  | ***K_m_*** | ***A_max_*** | ***K_m_*** | ***A_max_*** | ***K_m_*** | ***A_max_*** | ***K_m_*** | ***A_max_*** | ***K_m_*** | ***A_max_*** |
| **SP** | 0 | 0.41 ± 0.04 | 0.68 ± 0.02 | 0.20 ± 0.01 | 0.043 ± 0.0005 | 0.75 ± 0.04 | 0.117 ± 0.002 | 0.32 ± 0.02 | 0.0087 ± 0.0001 | 0.011 ± 0.001 | 0.0023 ± 0.0001 |
|  | 10 | 0.71 ± 0.05 | 0.72 ± 0.01 | 0.45 ± 0.04 | 0.043 ± 0.001 | 1.23 ± 0.05 | 0.109 ± 0.001 | 0.94 ± 0.16 | 0.0080 ± 0.0004 | 0.009 ± 0.002 | 0.0017 ± 0.0001 |
|  | 50 | 1.52 ± 0.12 | 0.71 ± 0.02 | 1.24 ± 0.07 | 0.041 ± 0.001 | 3.72 ± 0.50 | 0.109 ± 0.006 | 3.42 ± 0.21 | 0.0074 ± 0.0002 | 0.015 ± 0.003 | 0.0016 ± 0.0001 |
|  | 200 | 2.93 ± 2.00 | 0.45 ± 0.11 | 3.44 ± 0.47 | 0.038 ± 0.002 | 9.61 ± 0.70 | 0.106 ± 0.004 | - | - | 0.023 ± 0.003 | 0.0016 ± 0.00005 |
|  | 1000 | 11.52 ± 2.77 | 0.54 ± 0.07 | 5.24 ± 1.49 | 0.019 ± 0.002 | 46.68 ± 92.12 | 0.075 ± 0.123 | - | - | 0.073 ± 0.014 | 0.0014 ± 0.0001 |
|  | 5000 | - | - | - | - | - | - | - | - | 0.228 ± 0.027 | 0.0010 ± 0.00003 |
| **GP** | 0 | 0.41 ± 0.04 | 0.68 ± 0.02 | 0.20 ± 0.01 | 0.043 ± 0.0005 | 0.75 ± 0.04 | 0.117 ± 0.002 | 0.32 ± 0.02 | 0.0087 ± 0.0001 | 0.011 ± 0.001 | 0.0023 ± 0.0001 |
|  | 2 | 0.42 ± 0.05 | 0.67 ± 0.02 | 0.31 ± 0.03 | 0.045 ± 0.001 | 0.94 ± 0.09 | 0.105 ± 0.003 | 0.61 ± 0.10 | 0.0088 ± 0.0004 | 0.030 ± 0.005 | 0.0026 ± 0.0002 |
|  | 10 | 0.73 ± 0.05 | 0.76 ± 0.02 | 0.61 ± 0.05 | 0.045 ± 0.001 | 3.10 ± 0.15 | 0.131 ± 0.002 | 2.68 ± 0.38 | 0.0093 ± 0.0005 | 0.051 ± 0.011 | 0.0018 ± 0.0001 |
|  | 50 | 1.34 ± 0.15 | 0.79 ± 0.03 | 1.79 ± 0.13 | 0.044 ± 0.001 | 12.36 ± 1.57 | 0.139 ± 0.010 | 3.73 ± 0.26 | 0.0056 ± 0.0001 | 0.051 ± 0.008 | 0.0011 ± 0.00005 |
|  | 200 | 3.66 ± 0.52 | 0.86 ± 0.05 | 13.01 ± 3.57 | 0.066 ± 0.011 | 35.17 ± 7.58 | 0.103 ± 0.018 | - | - | 0.508 ± 0.091 | 0.0016 ± 0.0001 |
|  | 1000 | - | - | - | - | - | - | - | - | 1.440 ± 0.294 | 0.0013 ± 0.0001 |
| **AP** | 0 | 0.39 ± 0.04 | 0.67 ± 0.02 | 0.28 ± 0.02 | 0.041 ± 0.001 | 0.77 ± 0.05 | 0.106 ± 0.002 | 0.51 ± 0.04 | 0.0084 ± 0.0002 | 0.007 ± 0.001 | 0.0017 ± 0.0001 |
|  | 0.5 | 0.39 ± 0.04 | 0.70 ± 0.02 | 0.26 ± 0.02 | 0.040 ± 0.001 | 0.71 ± 0.04 | 0.101 ± 0.002 | 0.48 ± 0.04 | 0.0084 ± 0.0002 | 0.010 ± 0.003 | 0.0014 ± 0.0001 |
|  | 2 | 0.50 ± 0.03 | 0.72 ± 0.01 | 0.29 ± 0.02 | 0.042 ± 0.001 | 0.70 ± 0.04 | 0.096 ± 0.002 | 0.57 ± 0.04 | 0.0085 ± 0.0002 | 0.274 ± 0.140 | 0.0056 ± 0.0022 |
|  | 10 | 0.39 ± 0.03 | 0.62 ± 0.01 | 0.26 ± 0.02 | 0.042 ± 0.001 | 0.75 ± 0.06 | 0.098 ± 0.002 | 0.62 ± 0.08 | 0.0090 ± 0.0003 | 0.594 ± 0.312 | 0.0088 ± 0.0041 |
|  | 50 | 0.41 ± 0.03 | 0.67 ± 0.01 | 0.29 ± 0.02 | 0.042 ± 0.001 | 1.33 ± 0.09 | 0.109 ± 0.002 | 0.99 ± 0.05 | 0.0096 ± 0.0002 | - | - |
|  | 200 | 0.45 ± 0.04 | 0.70 ± 0.02 | 0.40 ± 0.02 | 0.045 ± 0.001 | 2.46 ± 0.13 | 0.116 ± 0.002 | 2.79 ± 0.77 | 0.0097 ± 0.0010 | - | - |
|  | 1000 | 0.55 ± 0.09 | 0.63 ± 0.03 | 1.10 ± 0.09 | 0.049 ± 0.001 | - | - | - | - | - | - |
|  | 5000 | 1.17 ± 0.27 | 0.61 ± 0.04 | 5.24 ± 1.49 | 0.039 ± 0.004 | - | - | - | - | - | - |
